# Impacts of alkaline on the defects property and crystallization kinetics in perovskite solar cells

**DOI:** 10.1038/s41467-019-09093-1

**Published:** 2019-03-07

**Authors:** Yihua Chen, Nengxu Li, Ligang Wang, Liang Li, Ziqi Xu, Haoyang Jiao, Pengfei Liu, Cheng Zhu, Huachao Zai, Mingzi Sun, Wei Zou, Shuai Zhang, Guichuan Xing, Xinfeng Liu, Jianpu Wang, Dongdong Li, Bolong Huang, Qi Chen, Huanping Zhou

**Affiliations:** 1 Key Laboratory for the Physics and Chemistry of Nanodevices, Beijing Key Laboratory for Theory and Technology of Advanced Battery Materials, Department of Materials Science and Engineering, College of Engineering, Peking University, Beijing, 100871 P. R. China; 2 School of Material Science and Engineering, Beijing Institute of Technology, Beijing, 100081 P. R. China; 3 Department of Applied Biology and Chemical Technology, Hong Kong Polytechnic University, Hong Kong, 999077 P. R. China; 4 Key Laboratory of Flexible Electronics & Institute of Advanced Materials, Jiangsu National Synergetic Innovation Center for Advanced Materials, Nanjing Tech University, Nanjing, 211816 P. R. China; 5 CAS Key Laboratory of Standardization and Measurement for Nanotechnology, CAS Center for Excellence in Nanoscience, National Center for Nanoscience and Technology, Beijing, 100190 P. R. China; 6 Joint Key Laboratory of the Ministry of Education, Institute of Applied Physics and Materials Engineering, University of Macau, Macau, 999078 P. R. China; 7 Shanghai Advanced Research Institute, Chinese Academy of Sciences, Shanghai, 201210 P. R. China

## Abstract

Further minimizing the defect state density in the semiconducting absorber is vital to boost the power conversion efficiency of solar cells approaching Shockley-Queisser limit. However, it lacks a general strategy to control the precursor chemistry for defects density reduction in the family of iodine based perovskite. Here the alkaline environment in precursor solution is carefully investigated as an effective parameter to suppress the incident iodine and affects the crystallization kinetics during film fabrication, via rationale adjustment of the alkalinity of additives. Especially, a ‘residual free’ weak alkaline is proposed not only to shrink the bandgap of the absorber by modulating the stoichiometry of organic cation, but also to improve the open circuit voltage in the resultant device. Consequently, the certified efficiency of 20.87% (Newport) is achieved with one of the smallest voltage deficits of 413 mV in the planar heterojunction perovskite solar cell.

## Abstract (web summary)

Defect density reduction is pertinent for halide perovskite solar cells but a universal strategy has not been exploited. Here Chen et al. show that by fine tuning the alkaline environment in precursor solution, they can greatly suppress defects density and obtain high certified efficiency of 20.87%.

## Introduction

Organic–inorganic hybrid perovskite materials have attracted broad attention^1^, owing to their low fabrication cost and intriguing optical and electronic properties^2–5^. Impressively, it witnessed the power conversion efficiency (PCE) of perovskite solar cells boosted from 3.8%^6^ to a certified 23.7% rapidly^7–10^. To enable a truly competitive PCE of perovskite solar cells as that of the most efficient inorganic counterpart, e.g., GaAs, it is required to further reduce their energy loss. This relies on the improved charge generation and mitigated non-radiative charge recombination within the complete device. With careful investigations on the absorber materials^11,12^, device configuration^13,14^, and relevant interfaces^15,16^, it is found that the intrinsic defect properties^17,18^ in perovskite films influence the charge dynamics in the devices significantly. Halide perovskites are generally regarded as relatively soft ionic solids, which are prone to contain point defects^3,19^ in polycrystalline films (e.g., vacancies, interstitials, and cation and anti-site substitutions). They often serve as the non-radiative recombination centers to affect the quantum efficiency of photoluminescence and deteriorate the photovoltaic performance^17,20^. Therefore, to assure the best power output by reducing energy and voltage loss, it is of great importance to gain profound understanding on the defect physics and chemistry, which results in defects elimination in perovskite films for efficient photo-carrier transport.

During the past few years, efforts have been attempted to probe the defects to unveil their nature. In the methylammonium lead iodide (MAPbI_3_) perovskite, it reported a variety of shallow native point defects, e.g., Pb vacancies (V_Pb_), MA vacancies (V_MA_), interstitials MA (MA_i_), etc^3^., and deep energy-level defects^2^ in forms of complex and derivatives, such as interstitials I (I_i_) and its derivatives^21,22^. In mixed perovskites, it reveals even more complicated defect physics, wherein formamidinium (FA)-related defect I_FA_ (the complexes of I_i_ and V_FA_) makes a transition at a Fermi level that sits deep within the bandgap^19^. Furthermore, defect formation is reported to be correlated to the processing conditions, wherein films grown under iodine-rich conditions exhibit a higher defect density^23^. Among all defects, iodine-related defects receive most interest. It is reported that polyiodide complexes in dimethylformamide (DMF) solution could easily induce defects due to a nonstoichiometric Pb/I ratio in the resultant films^24^. When dimethyl sulfoxide (DMSO) was further introduced, either I-rich or I-deficient intermediates were identified, depending on the ratio of the starting materials^25^. Recently, it demonstrated iodine redox chemistry to convert hole traps into electron traps possibly by oxidation during film processing^26^. Subsequently, the iodide management was proposed to effectively decrease the concentration of mid-gap defects in the perovskite films, and improve the PCEs of perovskite solar cells^27–29^. Yet, it lacks a feasible approach to be universally adopted in various halide perovskites, which suppresses the incidental I_2_ in the absorber for enhanced device performance with reduced energy and voltage loss.

In this work, we demonstrate a universal technique to suppress the incident I_2_ in the halide precursor solution by employing alkaline, wherein iodine can be effectively eliminated via a disproportionation reaction under a number of different alkaline environments. The alkalinity is further demonstrated to affect the crystallization kinetics and optoelectronic properties of perovskite films. For instance, formamidine acetate as a ‘residual free’ weak alkaline, is found to effectively manipulate the stoichiometry of the cations in the mixed perovskite ((FA,MA,Cs)Pb(I,Br)_3_) precursor, and in particular to reduce the density of deep-level defects in the resultant films substantially. Consequently, the corresponding mixed perovskite solar cells achieve an average PCE of 20.87% (certified). More interestingly, this modified absorber possesses a smaller bandgap, but the resultant device revealed unambiguous improvement in the open-circuit voltage (*V*_OC_), corresponding to a *V*_OC_ deficit of 413 mV, which is one of the smallest certified value in the planar perovskite solar cells. These findings provide a universal approach for defect reduction in iodide-based perovskite films, which substantially reduce the energy and voltage loss to boost the efficiency of the devices.

## Results

### Suppression of I_2_ impurity via alkaline additives

**Fig. 1:**
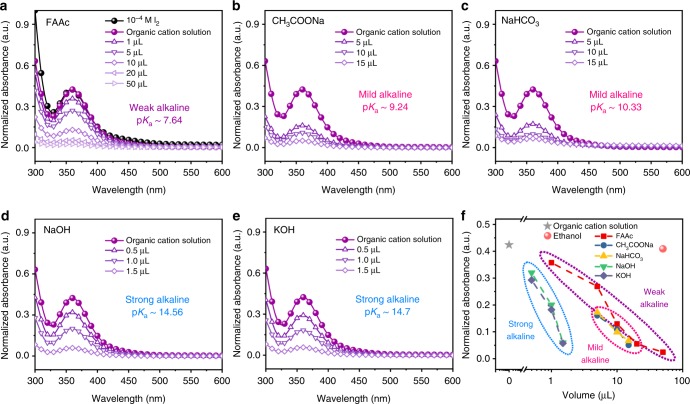
Suppression of I_2_ impurity via alkaline additives. UV-vis absorption spectra of the organic cation solution, which is used to prepare the perovskite films, with different amounts of (**a**) FAAc, (**b**) CH_3_COONa, (**c**) NaHCO_3_, (**d**) NaOH, and (**e**) KOH ethanol solution. **f** Normalized absorbance for organic cation solution with different additives as a function of the volume

Precise control of precursor solutions is crucial for perovskite film deposition. Generally, the organic precursors are hygroscopic and unstable in air or under illumination. In particular, formamidine iodide (FAI) and methylammonium iodide (MAI) can be easily oxidized to form I_2_ and/or other by-products, which induces the nonstoichiometric ratio in the precursor solution. It further leads to defect states in perovskite films that eventually deteriorate device performance. The degradation mechanism involves the formation of I_2_, which is thoroughly studied in previous work^30^. Ultraviolet-visible (UV-vis) absorption spectroscopy was conducted to semiquantitatively investigate the formation of iodide species in the precursor solution^27–29,31^. As shown in Fig. 1a, the organic cation solution used to prepare the perovskite films shows a characteristic peak at 360 nm. It is often attributed to the absorption of I_3_^−^, stemmed from I^−^ reacting reversibly with I_2_. Interestingly, the peak intensity equals to that of 10^−4^ M I_2_ in isopropanol, indicating the trace amount of I_2_ in the organic cation precursor solution.

To eliminate the incident I_2_ in the precursor solution, we propose to create an alkaline environment by introducing additives in the precursor solution. A series of alkaline additives with different alkalinity were employed, such as formamidine acetate (FAAc, p*K*_*a*_ ~7.64), sodium acetate (CH_3_COONa, p*K*_*a*_ ~9.24), sodium bicarbonate (NaHCO_3_, p*K*_*a*_ ~10.33), sodium hydroxide (NaOH, p*K*_*a*_ ~14.56), and potassium hydroxide (KOH, p*K*_*a*_ ~14.7), to create the full spectrum of alkalinity precursor environment in the context of the alkalinity (see Supplementary Notes 1 and 2 for details). Take the alkaline solution of FAAc/ethanol for example, we observed that along with an increasing amount of FAAc, the absorbance at 360 nm for precursor solution was dramatically reduced (Fig. 1a). Similarly, reduced absorbance at 360 nm was also observed when FAAc was added to the iodine solution (10^−4^ M, Supplementary Fig. 1). This indicates that the diminishing absorbance at 360 nm in the precursor solution is due to the reduction of incident I_2_. Furthermore, CH_3_COONa, NaHCO_3_, NaOH, and KOH took the same effects as expected when added to the precursor solution (Fig. 1b–e). Meanwhile, the addition of pure ethanol does not reduce the absorption peak of I_3_^−^ (Supplementary Fig. 2). The disappearance of I_3_^−^ (mostly related to I_2_) in the alkaline environment possibly suggests a disproportionation reaction in the precursor solution, wherein I_2_ is converted into iodate and iodide^32^ (see Supplementary Note 3). In addition (Fig. 1f), we found that the alkalinity of additives would largely influence the disproportionation reaction. It requires less amount of NaOH or KOH to eliminate the I_2_ peak of precursor solution than that of FAAc, CH_3_COONa, or NaHCO_3_, mostly because the former ones are ‘strong’ alkaline in the context of conventional inorganic chemistry.

### Morphology, phase, and carrier lifetime analysis

**Fig. 2:**
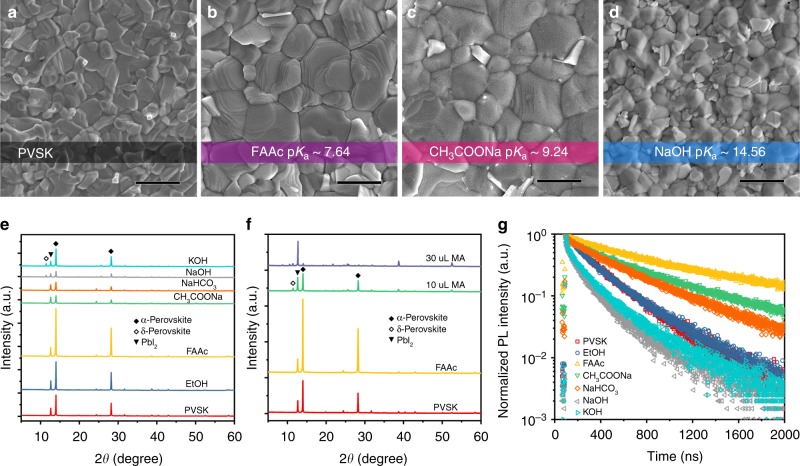
Morphology, phase, and carrier lifetime analysis. SEM images for the perovskite films deposited on SnO_2_-coated ITO glass prepared from the precursor solution with (**a**) no additive, (**b**) FAAc, (**c**) CH_3_COONa, and (**d**) NaOH ethanol solution, with a scale bar of 1 μm. **e** X-ray diffraction (XRD) pattern for the perovskite films deposited on SnO_2_-coated ITO glass prepared from the precursor solution with different alkaline additives. **f** XRD pattern for the perovskite films with NO additive, MA, and FAAc ethanol solution. **g** Time-resolved photoluminescence (TRPL) spectra for the perovskite films deposited on glass prepared from the precursor solution with different alkaline additives

The typical two-step approach was employed to fabricate the perovskite films (see details in the Methods section). We named the two-step processed reference perovskite film ((FA,MA,Cs)Pb(I,Br)_3_) as the PVSK film. To ascertain the impact of alkaline additives on the morphology and crystallographic structure of the resultant films, we performed scanning electron microscopy (SEM) and X-ray diffraction (XRD) measurements accordingly. Compared with the PVSK film, the perovskite films with CH_3_COONa or FAAc additive exhibited enlarged grain size (Fig. 2a–c), while those with NaOH or KOH remain the same grain size (Fig. 2d, Supplementary Fig. 3). It possibly indicates the different crystallization kinetics during film growth induced by the alkaline additives. Upon the employment of CH_3_COONa or FAAc, volatile acetate ions are reported to facilitate grain growth for better film quality^33^, which might account for the relatively enlarged grain size. On the contrary, perovskite films with the introduction of NaOH or KOH showed similar grain size, but a distinct crystalline grain morphology, as compared with the reference. It is often ascribed to the fact that Na^+^ or K^+^ is too small to incorporate into the perovskite lattice according to the Goldschmidt tolerance factor^34^. However, we cannot fully rule out the influences for crystal growth in the kinetics perspective, given the different chemical environments introduced by different alkalines, which will be discussed later.

Furthermore, XRD results showed a dominating α-phase perovskite in all films (Fig. 2e), indicating that all these additives do not impact the thermodynamics of crystal growth substantially. However, PbI_2_ conversion process was affected by additives, as inferred from the formation of δ-phase perovskite, as well as the residual PbI_2_. We purposely controlled the stoichiometry in all samples with a bit excessive of PbI_2_ for efficient passivation^35,36^ (see also Supplementary Note 4). We observed that perovskite films with mild alkaline additives (CH_3_COONa or NaHCO_3_) only exhibited a relatively stronger PbI_2_ peak, while a distinct δ-phase perovskite peak occurred when strong alkaline additives (NaOH or KOH) were added during film fabrication. It suggests that the alkalinity of the additives would affect the formation of the by-products, where the stronger alkaline would promote the formation of the δ-phase and suppress the formation of the α-phase perovskite as well. To confirm our speculation, MA (p*K*_*a*_ = 10.62)/ethanol was introduced into the precursor solution (Fig. 2f). We found a considerable amount of δ-phase perovskite in the resultant perovskite film. As predicted, the formation of the α-phase perovskite was suppressed substantially when the content of MA/ethanol was twice more. Interestingly, compared with the other additives, the weak alkaline FAAc resulted in the desirable phase quality, with significantly improved film crystallinity and less PbI_2_ content, as compared with that of the reference and other additives.

The intrinsic defects in perovskite films affect the photogenerated carrier behavior and device performance, which also would be affected by the alkalinity of the additives. We therefore conducted time-resolved photoluminescence (TRPL) analysis to study the dynamics of carrier recombination. The perovskite films were deposited on glass by using a precursor solution with different alkaline additives. The TRPL decay curves (Fig. 2g) were fitted with the bi-exponential rate law (Supplementary Table 1). We found that the perovskite films with addition of a weak alkaline possessed a longer carrier lifetime than those with the addition of a strong alkaline. The prolonged lifetime of perovskite films with a weak alkaline additive, compared with that of the PVSK film, was attributed to the suppression of incident I_2_ in precursor solution and the desired film features, e.g., morphology and phase. Among all samples with alkaline additives, the perovskite film with FAAc exhibited the longest lifetime for both fast and slow recombination (*τ*_1_ = 132.7 ns and *τ*_2_ = 802.2 ns), indicating its lowest non-radiative recombination centers. To be noted, the introduction of Na^+^ or K^+^ also could possibly deform the perovskite crystal structure, which may lead to shallow defect sites or off-bandgap defects in perovskite films^37–40^ (see Supplementary Note 5).

### Crystallization kinetics control via alkalinity

**Fig. 3:**
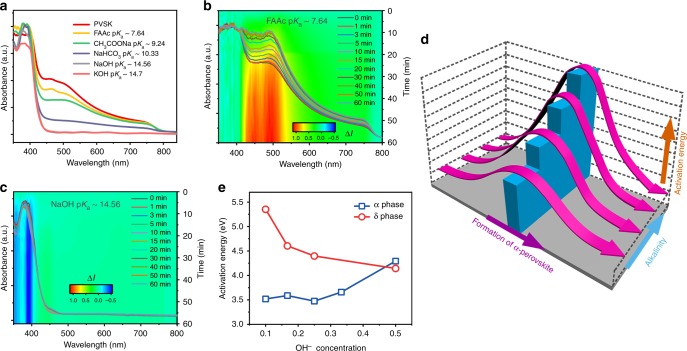
Crystallization kinetics control of perovskite films. **a** Real-time UV-vis absorption spectra for the perovskite films without annealing prepared under different alkalinity environments. In situ absorption measurement for perovskite films with addition of (**b**) FAAc or (**c**) NaOH. The inset figures are derived from the change of absorption intensity (∆*I*) for each sample at different times and the *y*-axis is the right one (Time). **d** Schematic illustration for the impacts of alkalinity on the crystallization kinetics of perovskite films. **e** Activation energies of the α- and δ-phase of FAPbI_3_ in the dependence of OH^−^ concentration

We further investigate the underlying crystallization kinetics of the formation of the α-phase perovskite by developing an in situ UV-vis absorption measurement. In a typical experiment, the organic cation solution was spin-coated onto the annealed PbI_2_ layer, which was further characterized by a UV-vis absorption spectrometer immediately. By varying the alkalinity in precursor solution (Fig. 3a), we clearly observed that the formation of the α-phase perovskite, whose main absorption peak ranged from 450 to 800 nm, was suppressed substantially when the alkalinity gets stronger. To be noted, the absorption before 450 nm might stem from the PbI_2_ or the δ-phase perovskite. Moreover, we judiciously selected two typical alkaline additives, e.g., FAAc (weak alkaline) and NaOH (strong alkaline), and continuously tracked the conversion evolution from PbI_2_ to the α-phase perovskite. Compared with the reference (Fig. 3b), the weak alkaline does not affect the formation of the α-phase perovskite, as expected. Accordingly, the absorption peak of the α-phase perovskite in the main range (450–800 nm) is increased continuously as time grows. The inset figure in Fig. 3b provides a visualization of the improved absorption in the range of 450 and 800 nm, suggesting the continuous formation of the α-phase perovskite. On the contrary (Fig. 3c), the formation of the α-phase perovskite with a strong alkaline was suppressed substantially. Even over time, only small changes occurred, as visualized in the inset figure of Fig. 3c. Based on the above analysis, we confirmed that the crystallization kinetics of the transformation from PbI_2_ to the α-phase perovskite, namely the activation energy, was influenced by the alkalinity of additives. As shown in scheme (Fig. 3d), when the alkalinity in the precursor gets stronger, the activation energy becomes larger, leading to an increased barrier for the transformation from the PbI_2_ to the α-phase perovskite. The results suggest that while incident I_2_ could be effectively eliminated via a disproportionation reaction under various alkaline environments, the alkalinity of the additives is also crucial to fabricate high-quality perovskite films with a desired phase. Because of the enrichment of the OH^−^ anion when alkalinity gets stronger, we speculate that the oxygen in the OH^−^ anion could participate in the coordination with lead iodide with stronger bonding. This may facilitate the structural transformation from PbI_2_ to the δ-phase perovskite, leading to an increased energy barrier for the transformation of PbI_2_ to the α-phase perovskite.

To verify the argument, we further performed the DFT simulation to analyze the exact activation energy for the formation of both the α-phase and δ-phase of the FAPbI_3_ perovskite in different conditions. The Calculation setup was given in Methods and Supplementary Note 6. As shown in Fig. 3e, two converse trends of the activation energy change are observed upon the introduction of OH^−^ into synthesis. At the low OH^−^ concentration, the δ-phase FAPbI_3_ has shown a much higher activation energy of 5.352 eV, which is 1.834 eV higher than that of the α-phase. As the OH^−^ concentration increases, the activation energies of δ-phase FAPbI_3_ decline from 5.352 to 4.143 eV. However, the activation energies of α-phase FAPbI_3_ increase from 3.518 to 4.293 eV, and finally exceed that of δ-phase FAPbI_3_. It clearly suggests that the formation of the α-phase perovskite is only preferred at a low OH^−^ concentration. With the increase of OH^−^ concentration, δ-phase FAPbI_3_ is more preferred kinetically, which is in consistent with the observation of the experiments above.

### Weak alkaline additives to reduce defect state density

To generalize the above findings, we selected three alkalines with a weak alkalinity, such as FAAc, triethanolamine hydrochloride (TEAHCl, p*K*_*a*_ ~7.8), and sodium hypophosphite (NaH_2_PO_2_, p*K*_*a*_ ~7.4), and incorporated them into different perovskite material systems, respectively. We conducted the TRPL measurement to characterize the carrier recombination profile of different perovskite films. The corresponding devices are subject to photovoltaic performance investigations (see Supplementary Note 7). The results demonstrate that the additives with a weak alkalinity could successfully decrease the trap state density of iodine-based perovskite films via effectively inhibiting incident I_2_ in the precursor solution. Combining the influence for alkalinity on the crystallization kinetics of perovskite films, the resultant devices show improved photovoltaic efficiency.

**Fig. 4:**
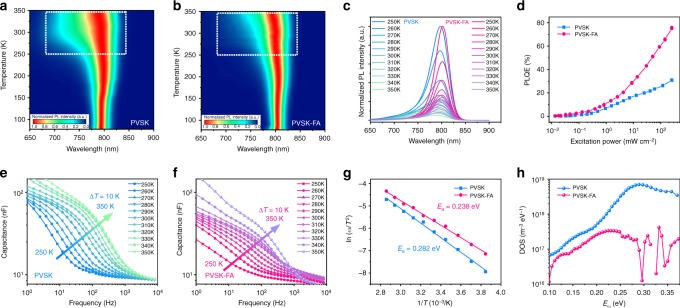
Weak alkaline additives to reduce defect state density. Color plots of normalized steady-state PL spectra for (**a**) PVSK and (**b**) PVSK–FA at different temperatures from 78 to 350 K. **c** Corresponding steady-state PL spectra for both films at various temperatures from 250 to 350 K. **d** The PL quantum efficiency of PVSK and PVSK–FA films as a function of excitation power. The admittance spectra of the (**e**) PVSK and (**f**) PVSK–FA devices measured at a temperature from 250 to 350 K with a step of 10 K. **g** The corresponding Arrhenius plots of the characteristic frequencies to extract the defect activation energy (*E*_a_) for both devices. **h** Trap density of states (tDOS) for both devices obtained by thermal admittance spectroscopy at room temperature

To obtain an in-depth understanding of the influence of a weak alkaline on the defect state of the perovskite, we chose the FAAc as an example and named the corresponding two-step processed perovskite film ((FA,MA,Cs)Pb(I,Br)_3_–FAAc) as the PVSK–FA. At first, steady-state photoluminescence (PL) spectra of PVSK and PVSK–FA over a temperature from 78 to 350 K were obtained and investigated (Fig. 4a, b). Within the temperature range of interest, the emission peak of PVSK–FA exhibited a distinct red shift as compared with PVSK, which was consistent with the UV-vis absorption results (Supplementary Fig. 4). It indicates a higher ratio of FA^+^ incorporated into the PVSK–FA due to the addition of an additive in the precursor. It was documented that the MAPbI_3_ film exhibited a broadening inhomogeneous multi-peak emission feature from a low-temperature phase, partially attributed to additional charge or exciton trap states only activated in low temperature^41^. However, in Figure 4a, b, we did not observe the above-mentioned multi-peak feature at a low temperature in the PVSK and PVSK–FA, indicating the characteristics of less traps. With temperature increasing, we observed a broadening of the emission line due to the electron–phonon coupling near room temperature, which was a consistent recent study in the FAPbI_3_ film^42^. Interestingly, the PL spectra of the PVSK–FA exhibited a better symmetry than the PVSK with temperature increasing from 250 to 350 K (Fig. 4c). On the one hand, the absence of an asymmetric feature in this film may suggest significant reduction of defect states near the band edge. It is believed that reduced grain boundaries may lead to reduced defects^43^, which is consistent with the morphology analysis of larger grains in the corresponding films (Fig. 2a, b). On the other hand, better symmetry in the PL peak indicates the absence of shoulder peaks often attributed to the slight phase segregation in the mixed perovskites^37^. It is thus implied that PVSK–FA possibly exhibited a retarded phase segregation and decreased defect states, when compared with the PVSK.

We further carefully examined the luminescence efficiency of the absorber, which serves as an important measure to identify a high-quality semiconductor^44–46^. Generally, deep-level defects serve as non-radiative recombination centers to capture the photogenerated charge carriers, thereby dramatically cutting down the quantum efficiency of photoluminescence and photovoltaic efficiency in solar cells. To be noted, a good solar cell has to be a good light-emitting diode^44^. According to Shockley–Queisser equation, the *V*_OC_ of the device decreases when the luminescence quantum efficiency of the absorber decreases. Here, the PL quantum efficiency (PLQE) of the perovskite film was examined as a function of excitation power (Fig. 4d). The PLQE was relatively small for either PVSK or PVSK–FA at low excitation power, but rapidly went up with the increase of excitation power. It indicates that the radiative recombination prevails at high optical injection levels, wherein non-radiative recombination centers were filled up, which have been further discussed in Supplementary Note 8. Notably, the PLQE of PVSK–FA achieved 61% when the excitation power was 100 mW cm^–2^ (close to the device working condition), which is more than twice as that of PVSK (PLQE = 25%). It further revealed obvious enhancement of PLQE compared with PVSK among the entire excitation power range, confirming the reduction of deep-level defect state in PVSK–FA. It correlates to the improvement in *V*_OC_ in the corresponding device, as will be discussed later.

To find the defect energy level in the perovskite films, we conducted thermal admittance spectroscopy (TAS) analysis. It is widely adopted in perovskite solar cells to extract the native defect state-level information through the identification of junction capacitance. Given a p-type perovskite semiconductor, the defect activation energy (*E*_a_) is approximately the depth of defect state energy level relative to the valance band of the perovskite^47,48^. Here, TAS measurement was carried out from 250 to 350 K with a step of 10 K to obtain the temperature-dependent admittance spectra for PVSK and PVSK–FA-based devices (Fig. 4e, f). The characteristic transition angular frequency (*ω*_0_) was extracted from the derivative of the capacitance–frequency spectrum. The relationship between *E*_a_ and *ω*_0_ follows the equation: *ω*_0_ = *βT*^2^ exp(−*E*_a_/*kT*), where *β* is a temperature-dependent parameter, *k* is the Boltzmann’s constant, and *T* is the temperature. The Arrhenius plot (Fig. 4g) derived from the temperature-dependent *ω*_0_ and fitted curves revealed that the *E*_a_ of PVSK and PVSK–FA devices were 0.282 and 0.238 eV, respectively.

Moreover, we attempted to illustrate the energetic profile regarding the trap density of states (tDOS) in the perovskite absorber (Fig. 4h). It could be derived from the equation *N*_T_(*E*_*ω*_) = − *ωV*_bi_/*qkTW* *×* d*C*/d*ω* and *E*_*ω*_ = *kT* ln(*βT*^2^/*ω*), where *q* is the elementary charge, *ω* is the angular frequency, *V*_bi_ is the built-in potential, and *W* is the depletion region, respectively^49^. The *V*_bi_ and *W* were obtained from the Mott–Schottky analysis (Supplementary Fig. 5). As expected, the PVSK device exhibited a relatively large density of defect states, especially around the deep defect level at 0.282 eV. Previous DFT calculation study^26^ reported that this defect-state level (0.282 eV) could potentially be ascribed to iodine interstitials (I_i_), which was the deep-level defect state and dominating non-radiative recombination centers in devices. Interestingly, the PVSK–FA device exhibited a striking reduction of the deep-level defect state at 0.282 eV for two orders of magnitude. In addition, the shallow defect states (0.1–0.2 eV) were also suppressed. Considering the analysis above, we reasonably claim that the introduction of a weak alkaline additive could effectively suppress the incident I_2_ in the precursor solution and significantly reduce the I_i_ defect states and achieve a much smaller defect activation energy in the resultant perovskite films. In this case, our iodine suppression approach looks contradictory with the previous research^29^; however, we argue that the discrepancy in these two approaches of iodine management is mostly due to different physiochemical properties in the corresponding absorber layers. It likely stemmed from the two film growth techniques applied and different material compositions, which is further discussed in Supplementary Note 9.

Beside of defects at the deep energy level, tail defects at the band edge were further investigated. Urbach tail profiles of perovskite films were calculated according to the formula equation: *α* = α_0_ exp(*hv*/*E*_U_), where α_0_ is a constant, *E*_U_ denotes Urbach energy, and *α* is an absorption coefficient. The absorption coefficient versus energy plots were presented in Supplementary Fig. 6. As is reported^33,50^, low *E*_U_ is highly desirable for semiconductor devices, indicative of less electronic disorders in the crystal lattice (or less impurities). Accordingly, the *E*_U_ values were estimated to be 31.22 and 29.64 meV for PVSK and PVSK–FA, respectively. Assuming the same level of thermal disorder, the smaller *E*_U_ value obtained from PVSK–FA demonstrated a lower level of electronic disorder at the band edge.

To quantitatively assess the concentration of defects across the entire device, the current density–voltage curves under the dark state for capacitor-like devices with indium tin oxide (ITO)/perovskite/gold (Au) configuration were measured (Supplementary Fig. 7). The linear current density–voltage relation at the low bias suggested an ohmic response, and the transition point was considered as the voltage at which all the traps were filled (trap-filled limit voltage: *V*_TFL_). The concentration of defects was determined by the equation *N*_defects_ = 2*εε*_*0*_*V*_TFL_/*qL*^2^, where *q* is the elementary charge, *ε* is the relative dielectric constant, *ε*_*0*_ is the vacuum permittivity, and *L* is the thickness of the perovskite films obtained from the cross-sectional SEM images of the ITO/perovskite/Au devices (Supplementary Fig. 8). It is reasonable to assume that the slight introduction of an additive would not affect the relative dielectric constants of perovskite films significantly^51^. The defect density *N*_defects_ was estimated to be 1.38 × 10^16^ and 0.47 × 10^16^ cm^−3^ for PVSK and PVSK–FA devices, respectively. It clearly confirms that the defect density in the perovskite absorber has been significantly reduced to nearly an order of magnitude by the addition of a weak alkaline additive. Moreover, we have conducted femtosecond optical measurements^52^ and confirmed that PVSK–FA exhibited a lower defect density than the PVSK film, which is the same order of magnitude as the above result (Supplementary Note 10).

### Weak alkaline additive to improve photovoltaic performance

**Fig. 5:**
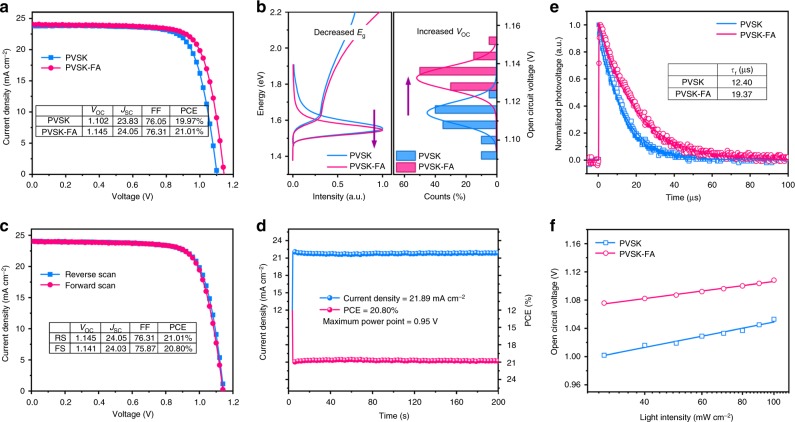
Photovoltaic device performance. **a** Current density–voltage curves for PVSK and PVSK–FA devices. **b** Left part: the UV-vis and PL spectra of for the PVSK and PVSK–FA films. Right part: the histogram of *V*_OC_ for the PVSK and PVSK–FA devices and the curve represents Gaussian function fit to the statistics data. **c** The champion device performance under reverse scan (1.2 to –0.1 V) and forward scan (–0.1 to 1.2 V), with a scan rate of 40 mV s^–1^. **d** Steady-state current density and power conversion efficiency holding the voltage at the maximum power point (0.95 V) for the PVSK–FA device. All these photovoltage characterizations were carried out under standard AM 1.5 radiation at ambient condition. **e** The transient photovoltage decay curves and (**f**) *V*_OC_ as a function of light intensity for the PVSK and PVSK–FA devices

The significant reduction in intrinsic defect density within the perovskite film was expected to suppress the non-radiative recombination channel effectively and improve the device performance. To prove it, we fabricated the planar heterojunction devices using the structure ITO/SnO_2_/perovskite/spiro-OMeTAD/Au (Supplementary Fig. 9) and studied the impacts of weak alkaline additives on photovoltaic performance. Figure 5a presents the current density–voltage (*J*–*V*) curves of the optimized perovskite devices measured under standard AM 1.5 G at ambient condition. The typical PVSK device yielded a PCE of 19.97% with a *V*_OC_ of 1.102 V, a short-circuit current density (*J*_SC_) of 23.83 mA cm^–2^, and a fill factor (FF) of 76.05%. Meanwhile, the best PVSK–FA device exhibited a significantly improved performance with a *V*_OC_ of 1.145 V, a *J*_SC_ of 24.05 mA cm^–2^, a FF of 76.31%, and the corresponding PCE of 21.01%. One of our best devices was sent to the accredited PV calibration laboratory (Newport, USA) and the certified PCE was 20.87% (Supplementary Fig. 10). The slightly enhanced *J*_SC_ value was attributed to improved light-harvesting capacity and a slightly red shift of the absorption onset. The major improvement in device performance was contributed from *V*_OC_ enhancement, thanks to the high-quality perovskite thin film with significantly reduced intrinsic defect density by the addition of a weak alkaline additive.

To be emphasized, in the present study, the addition of FAAc could slightly reduce the bandgap of the resultant film from 1.57 eV to 1.56 eV but increase *V*_OC_ from 1.102 to 1.147 V in the resulting device. It is interesting that although the bandgap of the perovskite film decreased, the *V*_OC_ of the relevant solar devices actually improved (Fig. 5b). It thus reveals one vital feature for this high-efficiency device, which is the small *V*_OC_ deficit (defined as *E*_g_/*q* −*V*_OC_, where *E*_g_ is the optical bandgap and *q* is the elemental charge). *V*_OC_ deficit is an essential indicator to measure the potential of the photovoltaic material and the maturity of the relevant technology. Current *V*_OC_ deficit for c-silicon, GaAs, and organic solar cells are around 400, 300, and 600 mV, respectively^53^. Surprisingly, the corresponding *V*_OC_ deficit in our device decreased to 413 mV upon FAAc addition, wherein both the *V*_OC_ (1.147 V) and the bandgap (1.56 eV) were certified through a third party. The *V*_OC_ deficit of 413 mV is one of the smallest certified value for planar heterojunction perovskite solar cells to date. It further implies substantially reduced non-radiative recombination losses in the PVSK–FA device, which was consistent with the high PLQE measured previously.

In addition, we did not observe an appreciable *J*–*V* hysteresis in the corresponding device, and the average PCE of 20.91% was achieved (Fig. 5c and Supplementary Fig. 10). The integrated photocurrent density gave a calculated value of 23.47 mA cm^−2^ from the external quantum efficiency (EQE) spectrum (Supplementary Fig. 11), which was comparable with the result from *J*–*V* measurement. In addition, a steady-state current density of 21.89 mA cm^−2^ and a stabilized PCE of 20.80%, were also achieved by holding the voltage at the maximum power point (0.95 V) for the PVSK–FA device (Fig. 5d). To probe the reliability and repeatability, 20 individual devices were fabricated with the identical processing condition and the corresponding statistical distribution of the PCE values was presented (Supplementary Fig. 12). The average efficiency for the PVSK and PVSK–FA devices were 19.34% and 20.63%, respectively. Furthermore, we have investigated the long-term stability of the PVSK and PVSK–FA-based devices, as shown in Supplementary Fig. 13. The reference without further encapsulation degraded to be around 80% within 1500 h of storage in an N_2_ glove box, whereas the PVSK–FA-based devices exhibited less degradation. It is possibly because of the higher thin-film quality of PVSK–FA with reduced defect states, which inhibits the penetration of undesirable water or oxygen molecules that further decompose perovskite films. Meanwhile, it may also correlate to the elimination of incident I_2_, which has been demonstrated to induce the chemical chain degradation of the perovskite^31^.

To gain further insights into the carrier dynamics across the device, we measured transient photocurrent (TPC) and photovoltage (TPV) decays of PVSK and PVSK–FA-based devices on the microsecond scale^54^. The PVSK–FA-based device exhibited a faster decay than that of the PVSK-based device, and the charge transport lifetime (*τ*_t_) derived from TPC measurements (Supplementary Fig. 14) was decreased from 4.13 to 1.97 μs, indicating a dramatic improvement of charge transport capability for the PVSK–FA-based device. The TPV curves were correlated to the carrier recombination rates under the open-circuit condition in the full cell. We observed that the PVSK–FA-based device demonstrated a much slower decay, when compared with that of the PVSK-based device (Fig. 5e). The charge recombination lifetime (*τ*_r_) derived from the TPV curves was significantly prolonged from 12.40 to 19.37 μs, suggesting that the carrier recombination was predominately prohibited in the device. It indicates that the prolonged lifetime constant *τ*_r_ contributed from substantially reduced defect density in the perovskite material by the addition of weak alkaline additives.

Furthermore, we measured the *V*_OC_ change as a function of illumination intensity to elucidate the carrier recombination mechanism during device operation. For both PVSK and PVSK–FA-based devices, the *V*_OC_ increased monotonically with incident light intensity (Fig. 5f). According to the equation *V*_OC_ = *nkT*/*q* ln(*I*_SC_/*I*_0_ + 1), the values of the ideality factor were calculated to be 1.51 and 1.02, respectively. Generally, the ideality factor derails from unity if Shockley–Read–Hall (SRH) recombination occurs^55^. In our study, the ideality factor for the PVSK–FA-based device showed a significant reduction from 1.51 to 1.02, when compared with that of the PVSK-based device. It demonstrates that trap-assisted SRH recombination was suppressed substantially due to the reduced intrinsic defect density, which was in good agreement with previous results.

## Discussion

In summary, we demonstrate a universal pathway to effectively suppress the incident I_2_ defects and manipulate the stoichiometry via the iodine disproportionation reaction under an alkaline environment. The alkalinity is proven to be a crucial parameter that greatly impacts the crystallization kinetics and optoelectronic properties of the perovskite films. A weak alkaline consisting of the FA^+^ cation was demonstrated to serve as a ‘residual’ free agent to suppress deep-level defect density in the mixed perovskites ((FA,MA,Cs)Pb(I,Br)_3_), which provides extra benefits in tuning the A-site stoichiometry and film morphology. Featured with a narrower bandgap of the absorber though, the device exhibited a significant enhancement on *V*_OC_. It observed one of the smallest *V*_OC_ deficits of 413 mV in a certified planar heterojunction perovskite solar cell with an average PCE of 20.87%. These findings would bridge the gap between precursor chemistry and deep-level defect behavior in the perovskite film, particularly the iodide-based perovskite, which guides the design of next-generation perovskite solar cells with an efficiency approaching the theoretical limit.

## Methods

### Materials

All the reagents and chemicals were used as received, including PbI_2_ (99.999%, Sigma Aldrich), CsI (99.90%, Aladdin Industrial Corporation), N,N-dimethylformamide (99.99%, Sigma Aldrich), dimethyl sulfoxide (99.50%, Sigma Aldrich), sodium acetate (AR, Aladdin Industrial Corporation), formamidine acetate (99%, Aladdin Industrial Corporation), sodium bicarbonate (AR, Aladdin Industrial Corporation), sodium hydroxide (AR, Aladdin Industrial Corporation), potassium hydroxide (AR, Aladdin Industrial Corporation), sodium hypophosphite (99%, Aladdin Industrial Corporation), triethanolamine hydrochloride (99.5%, Aladdin Industrial Corporation), spiro-OMeTAD (Xi’an Polymer Light Technology Corp.), chlorobenzene (99.9%, Aladdin Industrial Corporation), 4-tertbutylpyridine (99.90%, Sigma Aldrich), lithium bis(trifluoromethylsulfonyl)imide (99.95%, Sigma Aldrich), anhydrous ethyl alcohol (99.5%, Sigma Aldrich, product code: 459836), anhydrous isopropanol (99.5%, Sigma Aldrich, product code: 278475), and tin-doped indium oxide (ITO) substrates. All the formamidinium iodide (FAI), methylammonium iodide (MAI), methylammonium bromine (MABr), and methylammonium chloride (MACl) were synthesized and purified according to the procedure mentioned in the pervious literature^54^.

### Preparation of the SnO_2_ films

The tin-doped indium oxide (ITO) substrates were cleaned with acetone, deionized water, and isopropanol ultrasonically. The tin (IV) oxide colloidal dispersion (SnO_2_, 15% in H_2_O colloidal dispersion) was purchased from Alfa Aesar. The precursor solution was diluted by H_2_O to 2.67% and then spin-coated onto glass/ITO substrates at 4000 rpm for 30 s, and finally baked on a hot plate at 150 °C for 30 min in ambient air.

### Preparation of the perovskite precursor and films

To prepare the PbI_2_ and organic cation precursors, 600 mg of PbI_2_ and 18 mg of CsI were dissolved in 1 mL of mixture solvent of DMF:DMSO (9:1 v:v) and then annealed on a hot plate at 70 °C for 2 h with vigorous stirring. In all, 40 mg of FAI, 19 mg of MAI, 6 mg of MABr, and 5 mg of MACl were dissolved in 1 mL of isopropanol and then continuously stirred for 2 h at room temperature. To prepare the alkaline additive solution, 45 mg of different additives were dissolved in 1 mL of anhydrous ethyl alcohol at 70 °C for 30 min with vigorous stirring. Due to the different solubility of alkaline additives in ethyl alcohol, the possible supernatant was separated (Supplementary Note 11). For the certified perovskite solar cell, 45 mg of FAAc were dissolved in 1 mL of ethyl alcohol at 70 °C for 30 min with vigorous stirring. Then, 30 μL of FAAc ethanol solution was added to 1 mL of organic cation solution before being prepared with the perovskite film. The typical sequent-step method was employed to fabricate the perovskite film. First, the PbI_2_ precursor was spin-coated on the SnO_2_ substrate in an N_2_ glove box at 2300 rpm for 30 s (accelerated speed 6000 rpm s^–1^) and then annealed at 70 °C for 1 min in an N_2_ glove box. Then, the organic cation precursor was spin-coated on the PbI_2_ film at 2000 rpm for 30 s (accelerated speed 6000 rpm s^–1^) in an N_2_ glove box. Finally, the film was kept under vacuum for 5 min and annealed at 150 °C for 15 min in ambient air to form the perovskite film.

### Preparation of the Spiro-OMeTAD films and gold electrode

The Spiro-OMeTAD solution was first prepared by dissolving 72.3 mg of Spiro-OMeTAD, 17.5 μL of a stock solution of 520 mg mL^−1^ LiTFSI/acetonitrile, and 28.8 μL of tBP in 1 mL of chlorobenzene. Then the Spiro-OMeTAD precursor was spin-coated at 3000 rpm for 30 s (accelerated speed 6000 rpm s^–1^) in an N_2_ glove box. Finally, 100 -nm-thick gold was thermally evaporated as a counterelectrode under a pressure of 1 × 10^−4^ Pa on top of the Spiro-OMeTAD films to form the back contact by using a shadow mask to pattern the electrode.

### Calculation setup

The calculations have been applied through DFT within CASTEP packages^56^. For the functional, the Perdew–Burke–Ernzerhof-based generalized gradient approximation (GGA)^57^ has been selected to describe the electronic exchange–correlation interaction. All the geometry optimizations have been operated based on the Broyden–Fletcher–Goldfarb–Shannon (BFGS) algorithm, in which the residue of the Hellmann–Feynman forces will be converged to less than 0.001 eV A^–1^. The additional convergence criteria for the total energy and the interionic displacement are set to be less than 5 × 10^−5^ eV per atom and 0.005 Å per atom, respectively. The ultrafine plane-wave cutoff energy with the ultrasoft pseudopotential scheme has been chosen as the basis set. The OH^−^ molecule has been inserted into the PbI_2_ model and FAPbI_3_ unit cells with a different ratio n (*n* = 0.1–0.5) to represent the different concentration, which can be referred to as ‘#/unit cell’ in this simulation. The corresponding effect to the activation energies will be calculated based on the following equation:

1 $$E_{{\mathrm{act}}} = E_{{\mathrm{FAPbI}}_{{\mathrm{3}}\left( {{\mathrm{OH}}} \right){\mathrm{n}}}} - E_{{\mathrm{PbI}}_{{\mathrm{2}}\left( {{\mathrm{OH}}} \right){\mathrm{n}}}}-E_{{\mathrm{FAI}}}$$

The Monkhost–Pack reciprocal space integration was performed using a spacing of 0.07 Å^−1^ in all models with a different OH^−^ concentration^58^, which was also self-consistently suggested by total energy minimization. With this special k-point sampling, the total energy is converged within the maximum expected self-consistent cycles.

### Characterization

The absorbance of different precursor solutions and perovskite films was measured by a Hitachi UH4150 spectrophotometer. The integrating sphere was employed when we characterized the perovskite film. A field-emission SEM instrument (Hitachi S-4800) was used to acquire SEM images. The electron beam was accelerated at 5.0 kV. Temperature-dependent steady-state photoluminescence (PL) spectra were obtained by FLS980 (Edinburgh Instruments Ltd.), equipped with a Xe lamp, a liquid nitrogen cryostat (Oxford Instruments, OptistatDN-V), and a photomultiplier tube (PMT) detector. The excitation wavelength was 470 nm. The picosecond pulsed diode laser (EPL-470, Edinburgh Instruments Ltd.) was used to measure PL lifetime with a repetition rate of 0.2 MHz, a pulse width of 91.5 ps, a excitation fluence of ∼30 nJ cm^–2^, and maximum average power of 5 mW. The PLQE data were obtained from a three-step technique through the combination of a 445-nm continuous-wave laser, a spectrometer, an optical fiber, and an integrating sphere^59,60^. The powder XRD spectra (*θ*−2*θ* scans) were taken on a X’Pert3 powder X-ray diffraction system (PANalytical Inc.) with Cu Kα (λ = 1.5418 Å) as the X-ray source. The thermal admittance spectroscopy were conducted on Zahner Zennium pro Electrochemical Workstation at various temperatures (*T* = 250–350 K) in the dark from 10^0^ to 10^6^ Hz. A small AC voltage of 50 mV was used, and the DC bias was kept at zero to avoid the influence of the ferroelectric effect for perovskite materials during measurement. For temperature-dependent characterization, the sample was mounted in the Cryo Industries Liquid Nitrogen Dewars with Lake Shore model 335 cryogenic temperature controller. The current density–voltage (*J–V*) curves were measured (2400 Series SourceMeter, Keithley Instruments) under a simulated AM 1.5 G illumination at 100 mW cm^−2^ (Newport Thermal Oriel 91192 1000-W solar simulator). The light intensity was calibrated using a KG-5 Si diode. The *J–V* measurements were carried out in ambient air. The sweep speed was fixed at 40 mV s^–1^ (step 0.02 V, delay time 500 ms). The shading mask and one of our best device were sent to an accredited PV calibration laboratory (Newport, USA) for certification. The active area was defined as 0.09408 cm^2^. External quantum efficiencies (EQE) were obtained by an Enli Technology (Taiwan) EQE measurement system. A calibrated silicon diode with the known spectral response was used as a reference. For the transient photovoltage or photocurrent (TPV or TPC) decay measurements, a white light bias was from an array of diodes (Molex 180081–4320) to simulate 1-sun working condition, and the carriers were excited by a 532-nm pulse laser. The dynamics curves were recorded on a digital oscilloscope at a mega Ω (open-circuit condition for TPV) and a 50-Ω (short-circuit condition for TPC) resistor.

## Supplementary information

Supplementary Information

## Data Availability

The data that support the findings of this study are available from the corresponding author upon request.
