# Supplementary material for: Impacts of alkaline on the defects property and crystallization kinetics in perovskite solar cells
Source: Nat Commun. 2019 Mar 7;10:1112. doi: 10.1038/s41467-019-09093-1 (PMC6405758; doi:10.1038/s41467-019-09093-1)
Supplement: Supplementary file 1 — Supplementary Information [file 41467_2019_9093_MOESM1_ESM.pdf]

SUPPLEMENTARY INFORMATION

Impacts of Alkaline on the Defects Property and Crystallization Kinetics in Perovskite Solar Cells

Yihua Chen[1], Nengxu Li[1], Ligang Wang[1], Liang Li[1], Ziqi Xu[1], Haoyang Jiao[1], Pengfei Liu[2], Cheng Zhu[2], Huachao Zai[2], Mingzi Sun[3], Wei Zou[5], Shuai Zhang[6], Guichuan Xing[7], Xinfeng Liu[6], Jianpu Wang[5], Dongdong Li[4], Bolong Huang[3], Qi Chen[2] and Huanping Zhou[1]*

[1] Key Laboratory for the Physics and Chemistry of Nanodevices, Beijing Key Laboratory for Theory and Technology of Advanced Battery Materials, Department of Materials Science and Engineering, College of Engineering, Peking University, Beijing 100871, P. R. China.

[2] School of Material Science and Engineering, Beijing Institute of Technology, Beijing 100081, P. R. China.

[3] Department of Applied Biology and Chemical Technology, Hong Kong Polytechnic University, Hung Hom, Kowloon, Hong Kong, P. R. China.

[4] Shanghai Advanced Research Institute, Chinese Academy of Sciences, Shanghai 201210, P. R. China.

[5] Key Laboratory of Flexible Electronics & Institute of Advanced Materials, Jiangsu National Synergetic Innovation Center for Advanced Materials, Nanjing Tech University, Nanjing 211816, P. R. China.

[6] CAS Key Laboratory of Standardization and Measurement for Nanotechnology, CAS Center for Excellence in Nanoscience, National Center for Nanoscience and Technology, Beijing 100190, P. R. China.

[7] Joint Key Laboratory of the Ministry of Education, Institute of Applied Physics and Materials Engineering, University of Macau, Macau 999078, P. R. China.

*Corresponding author email: happy_zhou@pku.edu.cn

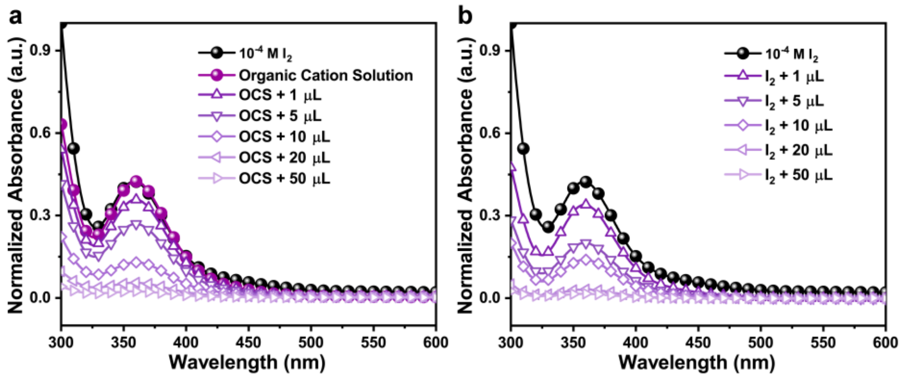

Supplementary Figure 1. UV-vis absorption spectra for (a) organic cation solution and (b) iodine solution (10^{-4} M) with different amount of FAAc ethanol solution.

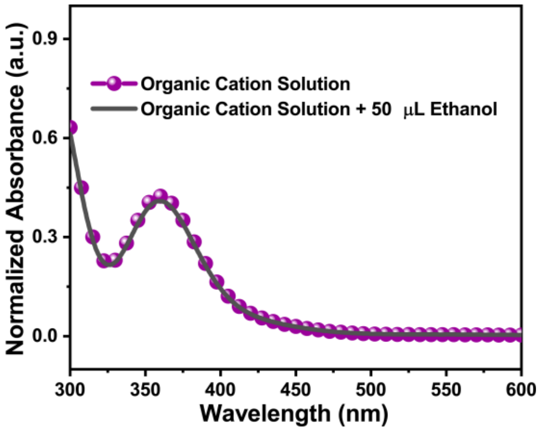

Supplementary Figure 2. UV-vis absorption spectra of organic cation solution with or without 50 μL pure ethanol.

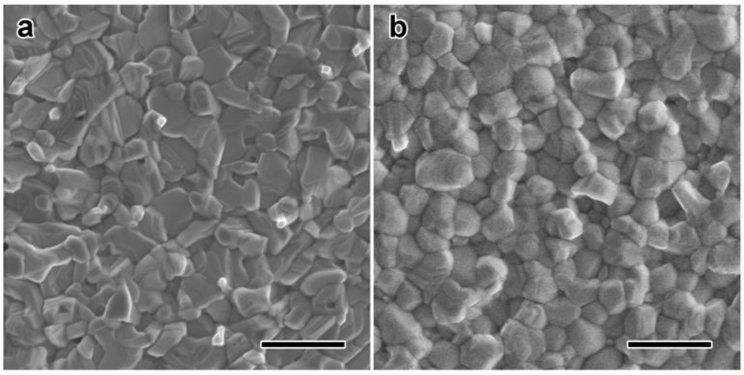

Supplementary Figure 3. SEM images for the perovskite films deposited on SnO_2 coated ITO glass prepared from the precursor solution with (a) no additive and (b) KOH ethanol solution, with scale bar of 1μm.

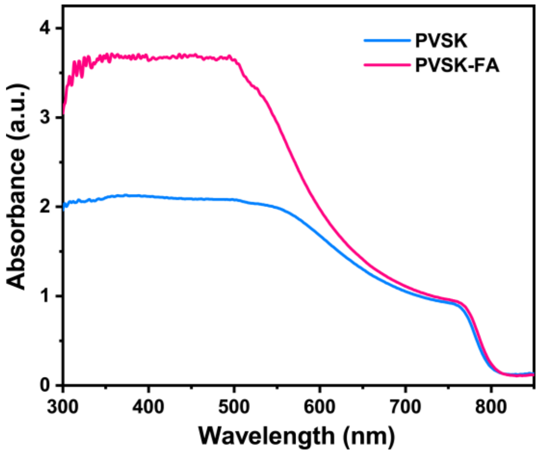

Supplementary Figure 4. The UV-vis absorption spectra for the PVSK and PVSK-FA films.

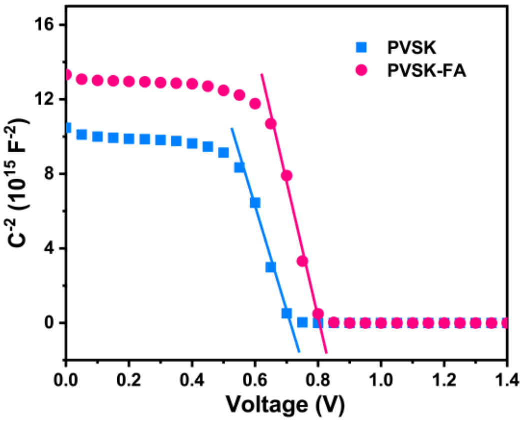

Supplementary Figure 5. Mott-Schottky analysis at 1 kHz for PVSK and PVSK-FA based devices.

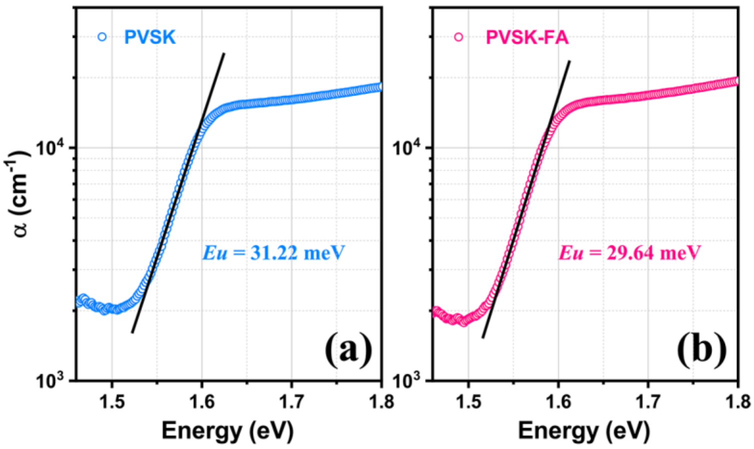

Supplementary Figure 6. Absorption coefficient derived from the UV-vis absorption spectra *versus* energy for (a) PVSK and (b) PVSK-FA films to calculate Urbach energies.

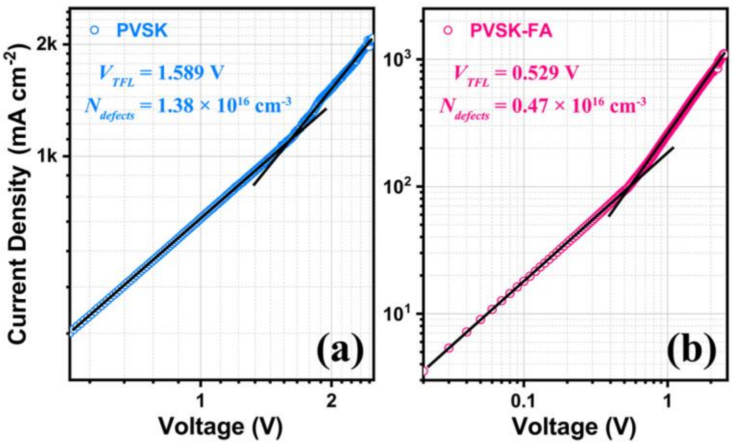

Supplementary Figure 7. Dark current density-voltage curves for devices with ITO/perovskite/Au configuration to estimate the defect density in PVSK and PVSK-FA films.

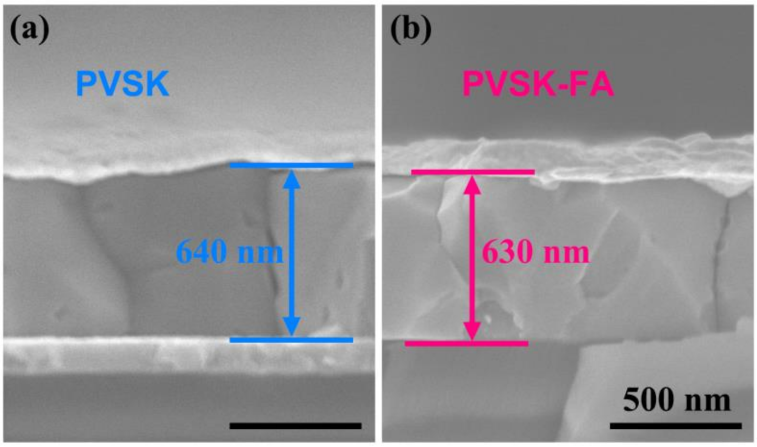

Supplementary Figure 8. The cross-sectional SEM images for the ITO/perovskite/Au devices, and the perovskite films were PVSK and PVSK-FA, respectively.

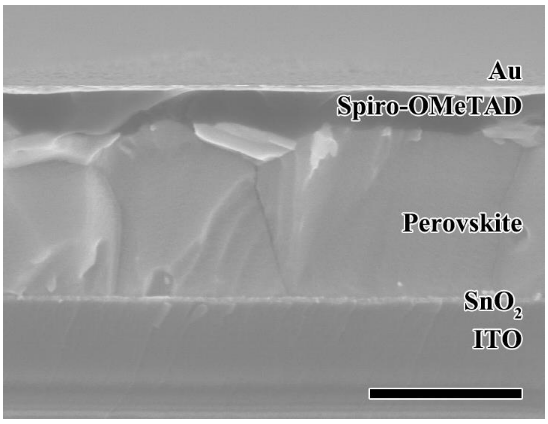

Supplementary Figure 9. The cross-sectional SEM image of the perovskite devices structured as ITO/SnO_2/perovskite/spiro-OMeTAD/Au, with scale bar of 500 nm.

Newport	A2LA ACCREDITED Calibration Cert. # 2893.01	**Technology and Application Center** **PV Lab** Newport Calibration Cert. # 1846

DUT S/N: 7164237 N157-1
Newport Calibration #: 1846
Manufacturer: Peking University College of Engineering and Beijing Institute of Technology
Material: Perovskite
Measurement Date: 2018-Feb-15
Temperature Sensor: TC-K, DUT Temperature: 25.0 ± 0.6 °C
Environmental conditions at the time of calibration: Temperature: 24 ± 3 °C; Humidity: 40 ± 10 %

The above DUT has been tested using the following methods to meet the ISO 17025 Standard by the PV Lab at Newport Corporation. Quoted uncertainties are expanded using a coverage factor of $k = 2$ and expressed with an approximately 95% level of confidence. Measurement of total irradiance is traceable to the World Radiometric Reference (WRR) and all other measurements and uncertainties are traceable to either NIST or CNRC and the International System of Units (SI). The performance parameters reported in this certificate apply only at the time of the test, and do not imply future performance.

Reported performance parameters are average of forward (short-circuit → open-circuit) and reverse (open-circuit → short-circuit) IV sweep results at a sweep rate of 40 mV/s, and do not represent the device behavior under stabilized conditions. At this sweep rate, hysteresis of +/- 0.25% of PCE was observed.

Efficency [%]	20.87	±	0.67	V_oc [V]	1.1464	±	0.0088	I_sc [A]	0.002237	±	0.000047
P_max [mW]	1.965	±	0.058	V_max [V]	0.964	±	0.014	I_max [A]	0.002038	±	0.000047
FF [%]	76.7	±	1.5	Area [cm^2]	0.0941	±	0.0005	M	0.985	±	0.015

Methods:
I-V: ASTM E948-16 *Standard Test Method for Electrical Performance of Photovoltaic Cells Using Reference Cells Under Simulated Sunlight*
QE: ASTM E1021-15 *Standard Test Method for Spectral Responsivity Measurements of Photovoltaic Devices*

Standard Reporting Conditions:
Spectrum: AM1.5-G (ASTM G173-03/IEC 60904-3 ed. 2)
1000.0 W/m^2 at 25.0°C

Secondary Reference Cell:
Device S/N: 10510-0054 KG1
Device Material: mono-Si
Window Material: KG1
Certification: National Renewable Energy Laboratory
A2LA accreditation certificate # 2236.01
ISO Tracking #: 1937
Certified short circuit current (I_{sc}) under standard reporting conditions (SRC): 56.87mA
Calibration due date: 29-Sep-19

Solar Simulator:
Spectrum: Newport Corporation filename *Sol3A_Spectroradiometer_Scan_0198.xls*
Total irradiance: 1000 W/m^2 based on I_{sc} of the above Secondary Reference Cell

Quantum Efficiency for DUT:
Newport Corporation filename *QE 1846, 7164237 N157-1, 0.43 mA WLB.log*
Spectral mismatch correction factor: M = 0.985 ± 0.015

DUT Calibration Procedures:
Newport Corporation document WI1 (EQE).docx
Newport Corporation document Area Measurement WI2 (Area).docx
Newport Corporation document WI3 (IV.Sweep).docx

Cal Cert V1.8	Issue Date: Feb 26, 2018	Page 2 of 2
	Reviewed and Approved by: Geoffrey Wicks	

Supplementary Figure 10. Certificated results from Newport Corporation PV laboratory. The certified efficiency is 20.87±0.67%. The mask area of 0.0941 cm^2 has been used.

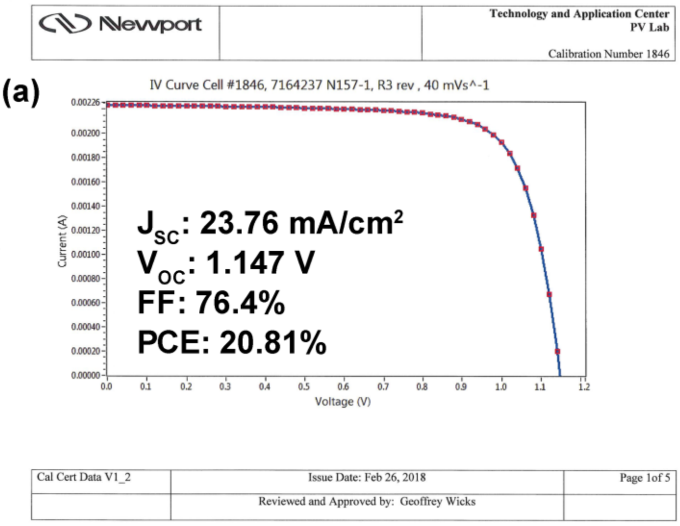

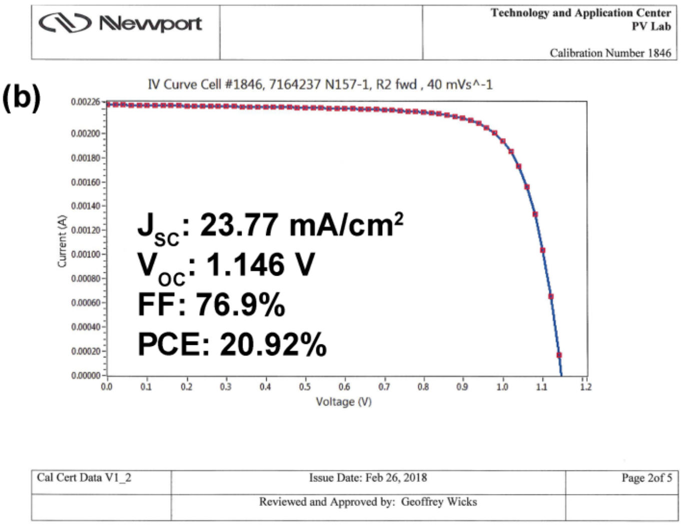

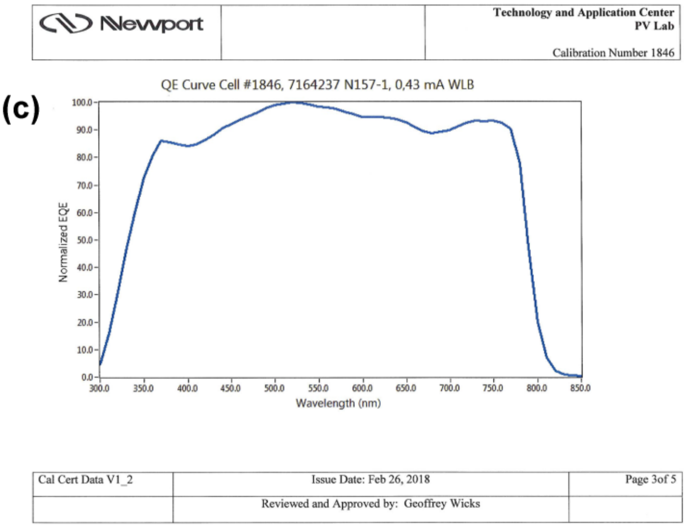

Supplementary Figure 10. Certificated results from Newport Corporation PV laboratory. (a) Reverse scan, (b) forward scan and (c) Normalized EQE.

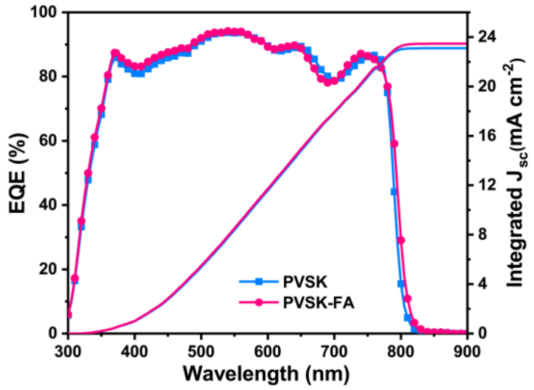

Supplementary Figure 11. The external quantum efficiency (EQE) spectrum and the corresponding integrated photocurrent density curve for PVSK and PVSK-FA devices.

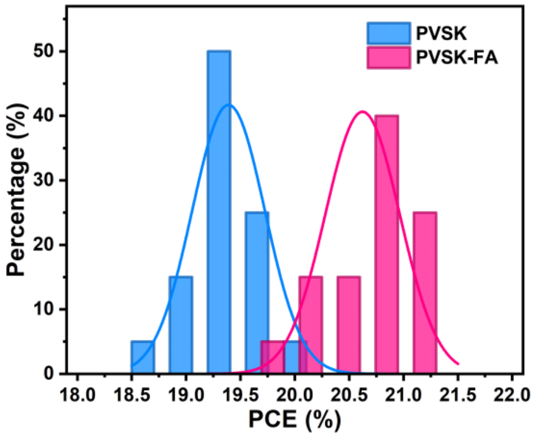

Supplementary Figure 12. Histogram of the PCEs for the PVSK and PVSK-FA devices, and the curve represents Gaussian function fit to the statistics data.

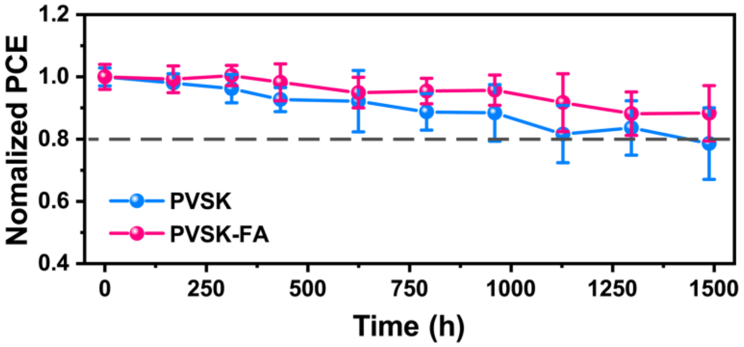

Supplementary Figure 13. The long-term stability of the PVSK and PVSK-FA devices stored in N_2 glove box, and the performance measurement was carried out in the ambient air with the humidity of 35-60%.

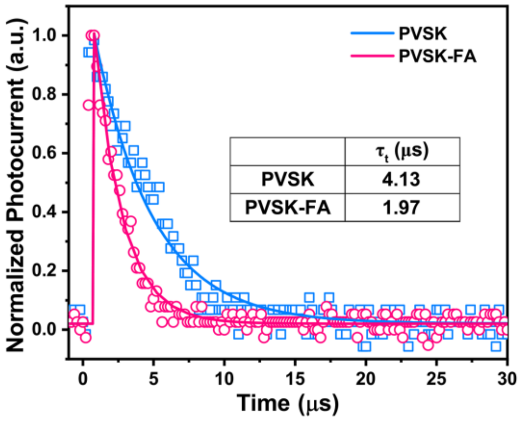

Supplementary Figure 14. The transient photocurrent decay curves for PVSK and PVSK-FA devices.

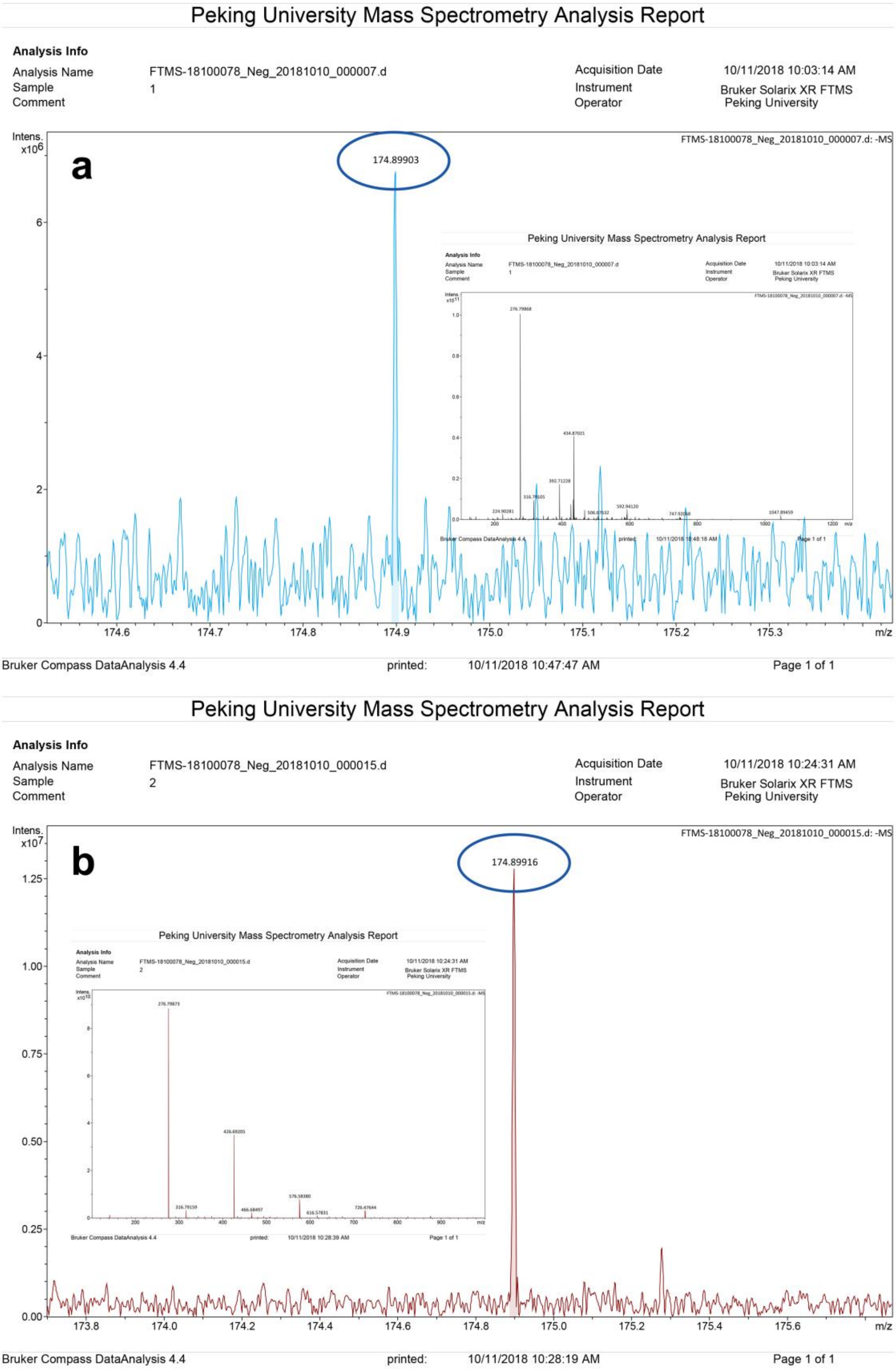

Supplementary Figure 15. Fourier transform ion cyclotron resonance mass spectra for I_2 isopropanol solution with addition of (a) NaOH aqueous solution and (b) NaOH ethanol solution.

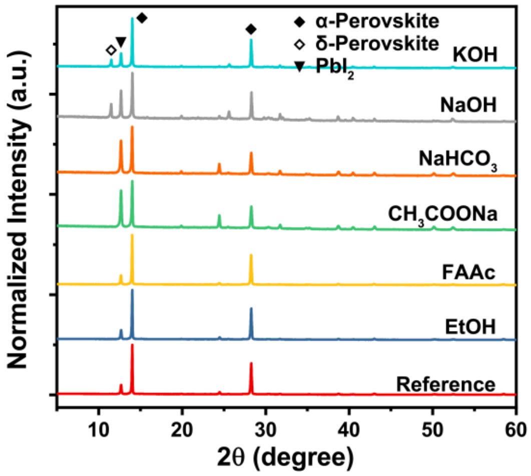

Supplementary Figure 16. The normalized X-ray diffraction (XRD) patterns for the perovskite films deposited on SnO_2 coated ITO glass prepared from the precursor solution with different alkaline additives.

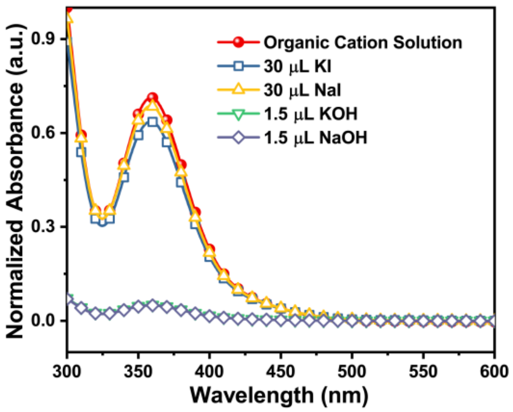

Supplementary Figure 17. UV-vis absorption spectra of the organic cation solution with different additives.

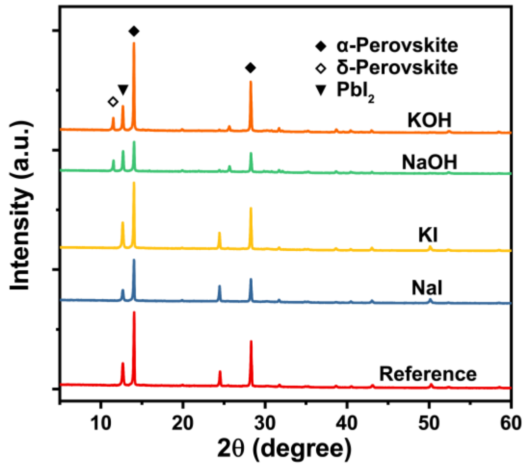

Supplementary Figure 18. X-ray diffraction (XRD) patterns for the perovskite films deposited on SnO_2 coated ITO glass prepared from the precursor solution with different additives.

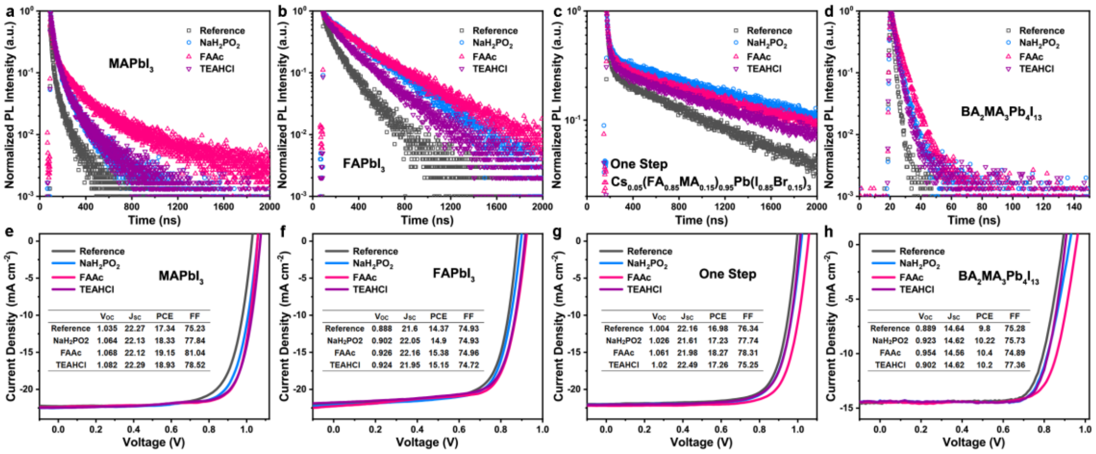

Supplementary Figure 19. The TRPL and *J*-*V* curves for the (a) two-step $MAPbI_3$, (b) two-step $FAPbI_3$, (c) one-step $Cs_{0.05}(MA_{0.85}FA_{0.15})_{0.95}Pb(I_{0.85}Br_{0.15})_3$ and (d) quasi two-dimensional $BA_2MA_3Pb_3I_{13}$ films and corresponding devices with addition of NO additive, NaH_2PO_2, FAAc or TEAHCl, respectively.

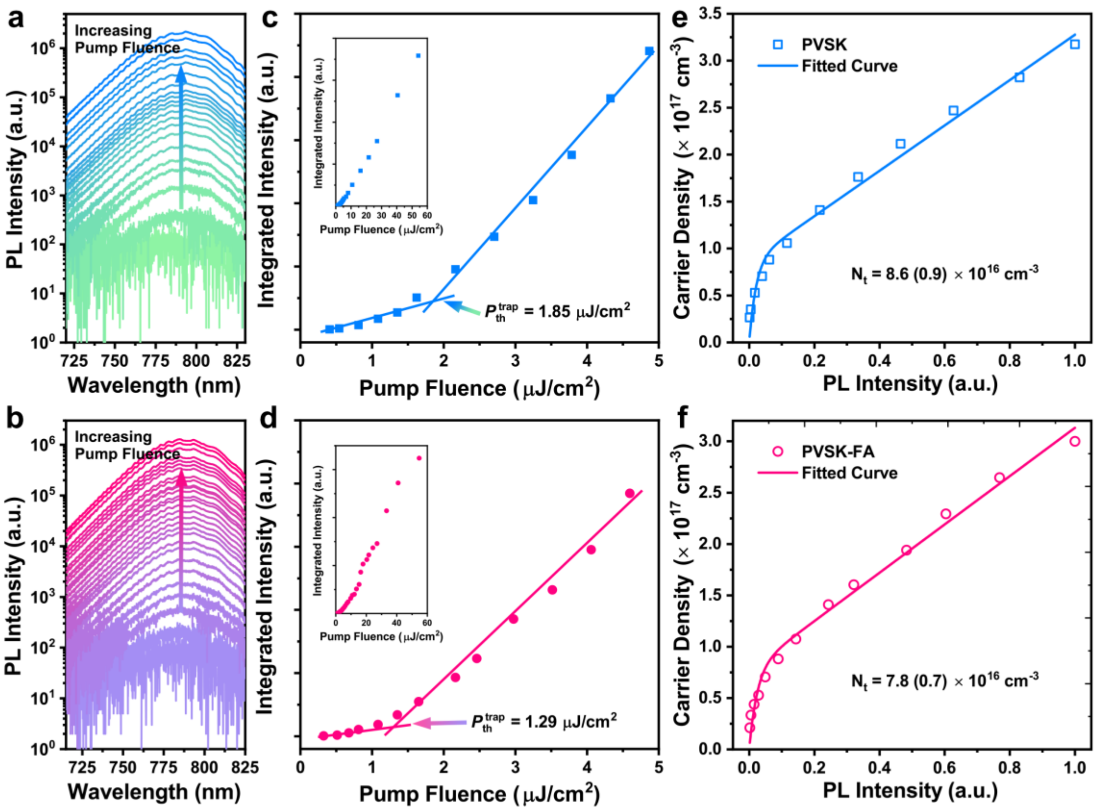

Supplementary Figure 20. Steady-state PL emission spectra photoexcited using 400 nm, 80 fs and 1 kHz pump pulses with increasing pump fluence for PVSK (a) and PVSK-FA (b) films. Integrated PL intensity as a function of pump fluence for PVSK (c) and PVSK-FA (d) films, which indicate the trap saturation threshold fluence (P_{th}^{trap}). The carrier density as a function of PL intensity within the low pump fluence range for PVSK (e) and PVSK-FA (f) films.

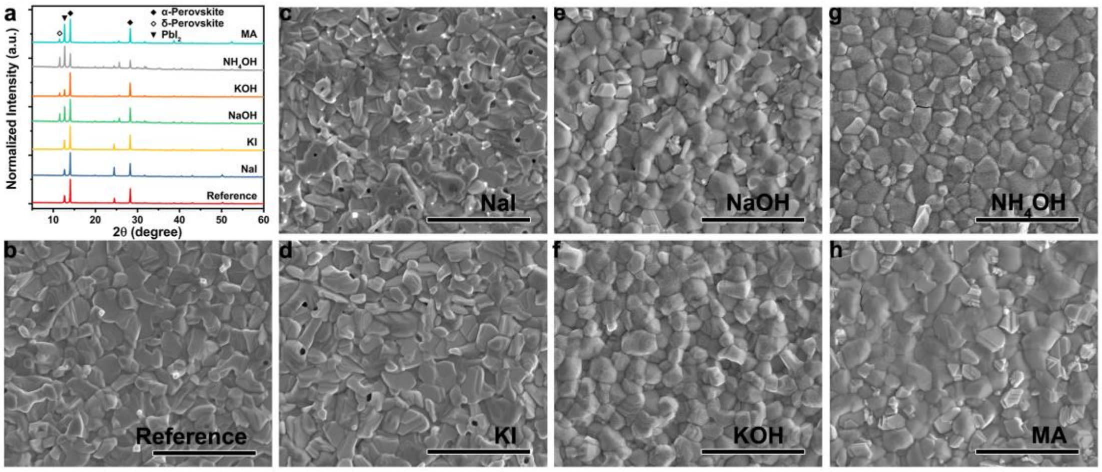

Supplementary Figure 21. The XRD patterns (a) and SEM images (b-h) of perovskite films with different alkaline additives.

Supplementary Table 1. The lifetimes extracted from the TRPL spectra of perovskite films with different alkaline additives.

	PVSK	EtOH	FAAc	CH_3COONa	$NaHCO_3$	NaOH	KOH
**τ1 (ns)**	108.0	99.5	132.7	38.2	105.9	26.2	23.2
**τ2 (ns)**	307.8	319.3	802.2	554.3	494.2	185.4	216.2

Supplementary Table 2. The definition of weak, mild or strong alkalinity for different additives.

	**pK_a**	**Phase State of Perovskite**	**Examples**
Weak	p$K_a \leq 8.5$	No influence for phase state	FAAc
Mild	$8.5 <$ p$K_a < 10.5$	The relatively increased PbI_2	CH_3COONa, $NaHCO_3$
Strong	p$K_a \geq 10.5$	The presence of δ-phase perovskite	NaOH, KOH

Supplementary Table 3. The lifetimes extracted from the TRPL spectra for different perovskite films with weak alkaline additives.

		**$MAPbI_3$**	**$FAPbI_3$**	**One-step#**	**$BA_2MA_3Pb_3I_{13}$**
**PVSK**	**τ1 (ns)**	24.3	23.4	15.8	1.43
	**τ2 (ns)**	152.5	117.2	864.7	9.14
**NaH_2PO_2**	**τ1 (ns)**	29.4	30.9	21.3	3.19
	**τ2 (ns)**	310.7	156.7	1341.7	18.22
**FAAc**	**τ1 (ns)**	29.8	42.9	23.1	4.27
	**τ2 (ns)**	344.5	285.9	1241.1	11.61
**TEAHCl**	**τ1 (ns)**	41.6	32.1	18.4	2.72
	**τ2 (ns)**	246.5	177.1	1129.4	16.59

One-step#: one-step processing $Cs_{0.05}(MA_{0.85}FA_{0.15})_{0.95}Pb(I_{0.85}Br_{0.15})_3$ film.

Supplementary Table 4. The solubility of alkaline additives in ethanol and the molar equivalence being added in the organic cation/isopropanol.

**Alkaline additives**	**Solubility in ethanol (mg)**	**Molar mass (mg/mmol)**	**Molar equivalence (10^{-4} mmol)**
**FAAc**	~ 45	104	~ 129.9
**CH_3COONa**	~ 7	82	~ 25.62
**$NaHCO_3$**	~ 1	84	~ 3.57
**NaOH**	> 45	40	> 337.5
**KOH**	> 45	56	> 241.2

Supplementary Note 1.

Several alkaline additives were selected in our experiment due to their different alkalinity, such as formamidine acetate (FAAc, pK_a ~ 7.64), sodium acetate (CH_3COONa, pK_a ~ 9.24), sodium bicarbonate ($NaHCO_3$, pK_a ~ 10.33), sodium hydroxide (NaOH, pK_a ~ 14.56) and potassium hydroxide (KOH, pK_a ~ 14.7). We must emphasize that the value of pK_a for different additives was measured in aqueous solution, wherein these ionic compounds could ionize completely. In this study, these alkaline additives were dissolved in ethanol solution, which could lead to the huge difference in the alkalinity of solution from that dissolved in aqueous solution due to due to the various solubility and dielectric environment. Thus, we could not determinate the exact alkalinity of these alkaline additives dissolved in ethanol solution. However, we speculate the alkalinity of these alkaline additives in this study was all based on the ethanol solution, which allow us to compare the alkalinity of various additives in ethanol by pK_a from the side view. The later results, including experiment and theoretical calculation, were consistent with this speculation about alkalinity of additives.

Supplementary Note 2.

To be more readable, we consider the whole experiment and theoretical calculation, and defined the meaning of weak, mild or strong alkalinity for different alkaline additives by pK_a and immediately related to the phase state of the resultant perovskite films, as listed in the Supplementary Table 2. The weak alkalinity would not affect the phase state of perovskite films. The mild alkalinity could influence the transformation from PbI_2 to δ-phase perovskite, appearing as the relatively increased PbI_2 content. The strong alkalinity could promote the formation of δ-phase and suppress the formation of α-phase perovskite in resultant film, appearing as the presence of δ-phase perovskite. This definition has both considered the pK_a and solubility difference between different alkaline additives. All detailed discussion will be given in the later.

Supplementary Note 3.

Iodine disproportionation reaction have been widely studied in environmental, nuclear and general chemistry[1]. Currently, most studies about iodine disproportionation reaction were based on the aqueous solution. To further investigate if the underlying equilibrium of iodine disproportionation in non-aqueous solution was same as that in aqueous solution, we employed Fourier transform ion cyclotron resonance mass spectrometry analysis to exam the possible iodate anion in the equilibrium. As shown in Supplementary Fig. 15, the mass spectrometers work in negative ion mode, and we observed the nearly identical characteristic peak around mass-to-charge (m/z) = 174.899 in both aqueous (Supplementary Fig. 15a) and non-aqueous solution (Supplementary Fig. 15b), which probably represent the existence of IO_3^- anion. This result indicated that the underlying equilibrium of iodine disproportionation in non-aqueous solution probably is same as that in aqueous solution and could been described as follows:

$$3I_2 + 6\mathbf{X} + 3H_2O \rightarrow 5I^- + IO_3^- + 6\mathbf{X}{\cdot}H^+ \quad (1)$$

wherein **X** refers to the base, and the trace amount of water comes from the solvent (ethanol and isopropanol).

Based on the above-mentioned equilibrium, the number of iodates was not significant in this reaction, even less than that of I_2. It is reported that incident I_2 have been found to induce the chemical chain degradation of perovskite films[2], but it does not apply to iodates. Thus, we think the effect of iodate ion in the system could be ignored.

Supplementary Note 4.

When we struggled for the certification of our high efficiency perovskite solar cells, we have studied the influence of the moderate amount of PbI_2 in perovskite solar cell. Just like other work that have been published[3,4], we agree the moderate amount of PbI_2 could effectively passivate the defect state in perovskite film and improve the device performance. Thus, our control sample contained significant fraction of PbI_2. To further help the readers exam the relative intensity of PbI_2 peak, we have normalized the XRD result in Fig. 2e (main text) as shown in Supplementary Fig. 16. We observed that perovskite films with CH_3COONa or $NaHCO_3$ additives showed obvious relatively stronger PbI_2 peak. Moreover, when NaOH or KOH additives were added into the system, the distinct δ-phase perovskite peak occurred. It suggested that the alkalinity of the additives would affect the transformation process from PbI_2 to α-phase perovskite.

Supplementary Note 5.

Considering introduction of Na^+ and K^+ and the Goldschmidt tolerance factor, it is reasonable to correlate the shallow defects sites due to deformed crystal structure. Meanwhile, many literatures[5,6] declared that the incorporation of Na^+ or K^+ would effectively reduce the defect density of perovskite film. These results indicate the introduction of Na^+ and K^+ actually helps to prevent at least deep energy level defects. In addition, our recent results also show that the cation cascade doping will guide the perovskite crystal orientation[7], which serves as one of major reasons for the improvement of device performance.

However, almost all of these studies above were based on the one-step approach, which was very different from the two-step method that we employed in the current study. In addition, when exploring the influence of NaI or KI on the iodine-based perovskite film, the iodine ion was always negligible, which is reasonable. However, in our experiment, the effect of OH^- is non-negligible and significant. Moreover, we don't deny the possible side effects of Na^+ or K^+ from NaOH or KOH on perovskite film. Besides these side effects, the major focus in our research is to reveal the significant effect of non-negligible OH^- induced by the alkaline additives on the precursor solution and resultant films. To clearly illustrate the different influence from anions, we further studied additives of NaI, KI, NaOH, and KOH. We conducted UV-vis absorption and XRD analysis, and the results are as shown in Supplementary Fig. 17 and 18. As expected, the NaOH and KOH additives could eliminate the incident I_2 (absorbance at 360 nm) in the precursor solution effectively, but NaI and KI could not. It clearly illuminates the effect of OH^- on suppression of I_2. Moreover, the NaI and KI incorporation have negligible influence on the crystallinity and phase of perovskite films, but NaOH and KOH additives would induce the formation of δ-phase and suppress the formation of α-phase perovskite. All these results have confirmed the significant role of OH^-.

Supplementary Note 6.

We further analyze the underlying film growth mechanism in a theoretical point of view. And we assume the same interdiffusion process between the organic and inorganic species for either the α-phase (cubic) and δ-phase (hexagonal) of $FAPbI_3$ reasonably, due to the same preparation method. The conversion process might be influenced by the alkalinity of additives. And we calculate the activation energies with existence of OH^- ions in the synthesis of the α-phase (cubic) and δ-phase (hexagonal) of $FAPbI_3$. The formation energies of both phase in normal condition and alkaline condition have been considered and the corresponding reactions of $FAPbI_3$ are shown as follows:

$$PbI_2 + FAI \rightarrow FAPbI_3 \quad (2)$$

$$PbI_2(OH)_n + FAI \rightarrow FAPbI_3(OH)_n \quad (3)$$

where the constant n represents the concentration of OH^- ions in the synthesis. The OH^- molecule has been inserted into the PbI_2 model and $FAPbI_3$ unit cells with different ratio n (n = 0.1 ~ 0.5) to represent the different concentration, which can be referred as "#/unit cell" in this simulation. The formation energies in normal condition of α- and δ-phase of $FAPbI_3$ have been calculated as 0.157 eV and -0.271 eV, respectively. Such results indicate δ-phase of $FAPbI_3$ is thermodynamically favored in normal synthesis conditions, in which the combination of both phase in the corresponding thin film is likely to be observed. After the introduction of OH^- during film formation, the activation energy changes for the two phases follow different patterns, as is summarized in Fig. 3e (main text). At the low OH^- concentration, the δ-phase $FAPbI_3$ has shown much higher activation energy of 5.352 eV, which is 1.834 eV higher than that of α-phase. As the OH^- concentration increases, the activation energies of δ-phase $FAPbI_3$ will experience an evident drop around 0.7 eV meanwhile α-phase $FAPbI_3$ will undergo a subtle increase (<0.1 eV) of the activation energies. Notably, the variations of activation energy of α-phase $FAPbI_3$ are relatively inert to the OH^- concentration at low level. On contrary, the activation energy of δ-phase $FAPbI_3$ is very sensitive, which decrease around 1 eV from n = 0.1 to n = 0.33 that is 10 times to the variation of α-phase $FAPbI_3$. Nevertheless, when the OH^- concentration reaches a high level (n = 0.5), the activation energy of these two phases will meet a cross, in which the δ-phase $FAPbI_3$ will keep decreasing to 4.143 eV. The α-phase $FAPbI_3$ will suddenly experience an obvious increase from 3.660 eV to 4.293 eV, which becomes larger than that of δ-phase $FAPbI_3$. All the above-mentioned results suggest that the OH^- concentration exhibits substantial influence to the formation of two phases of $FAPbI_3$. As the OH^- concentration increases, the formation of α-phase will gradually lose its advantage, and finally will be suppressed by the formation of δ-phase $FAPbI_3$ at high OH^- concentration. This preliminary result is in consistent with the observation of experiment above, illustrating the importance of control of OH^- in the synthesis of different phase of $FAPbI_3$.

Supplementary Note 7.

In this part, we aim to generalize our findings from two perspectives. Firstly, we verify that the above finding could be applied to all iodine-based perovskite films, e.g. $MAPbI_3$ and $FAPbI_3$, since the elimination of incident I_2 in organic precursor solutions could effectively reduce the defect state density in resultant films. Thus, four most widely studied perovskites with different processing are investigated, like one-step $Cs_{0.05}(MA_{0.85}FA_{0.15})_{0.95}Pb(I_{0.85}Br_{0.15})_3$, two-step $MAPbI_3$, two-step $FAPbI_3$, and quasi two-dimensional (2D) $BA_2MA_3PbI_{13}$ perovskite films. Secondly, we validate the operational guideline of weak alkalinity for additive selection by using formamidine acetate (FAAc, pKa ~ 7.64), triethanolamine hydrochloride (TEAHCl, pK_a ~ 7.8) and sodium hypophosphite (NaH_2PO_2, pK_a ~ 7.4) as examples.

Subsequently, we conducted the TRPL measurement to characterize the defect state profile of these different kinds of perovskite films with different additives. When TEAHCl, NaH_2PO_2 and FAAc were introduced into two-step $MAPbI_3$ (Supplementary Fig. 19a) film, the carrier lifetime was all prolonged than that of the reference film, suggesting the reduced defect state density. The reference film in this comparison is pure $MAPbI_3$ with NO additive. Similarly, when TEAHCl, NaH_2PO_2 and FAAc were introduced into two-step $FAPbI_3$ (Supplementary Fig. 19b) film, the carrier lifetime was all prolonged than that of the reference film, suggesting the reduced defect state density. The reference film in this comparison is pure $FAPbI_3$ with NO additive. We validated the effectiveness in one-step $Cs_{0.05}(MA_{0.85}FA_{0.15})_{0.95}Pb(I_{0.85}Br_{0.15})_3$ (Supplementary Fig. 19c) and quasi 2D $BA_2MA_3PbI_{13}$ (Supplementary Fig. 19d) films, respectively. The fitted lifetime values with the bi-exponential rate law also confirm this phenomenon (Supplementary Table 3).

We have further investigated the device performance based on the above different perovskite absorbers and additives, to find the relationship between TRPL and solar cell efficiency. As shown in Supplementary Fig. 19e, when TEAHCl, NaH_2PO_2 and FAAc were introduced into two-step $MAPbI_3$ device, the V_{OC} and efficiency show slight improvement than that of reference device, which is consistent with TRPL result (Supplementary Fig. 19a). The reference device in this comparison is pure $MAPbI_3$ device with NO additive. And similarly, as shown in Supplementary Fig. 19f, when TEAHCl, NaH_2PO_2 and FAAc were introduced into two-step $FAPbI_3$ device, the V_{OC} and efficiency also increased, consistent with TRPL result (Supplementary Fig. 19b). So it does in one-step $Cs_{0.05}(MA_{0.85}FA_{0.15})_{0.95}Pb(I_{0.85}Br_{0.15})_3$ (Supplementary Fig. 19g) and quasi two dimensional $BA_2MA_3PbI_{13}$ (Supplementary Fig. 19h) based devices, respectively.

To wrap up, we have validated the generality of our findings in both perspectives of additives and materials systems.

Supplementary Note 8.

The PLQE was relatively small for either PVSK or PVSK-FA at low excitation power, but rapidly went up with the increase of excitation power, which is consistent with other studies[8,9]. At the lower exciton power, the number of photon generated carriers is relatively small, which is trapped by the defect states leading to predominately non-radiative recombination. Thus, the PLQE of PVSK and PVSK-FA films are both very small. However, with the increase of exciton power, non-radiative recombination centers are gradually filled up and the bimolecular radiative recombination became dominant. Thus, the PLQE was increased dramatically for both PVSK and PVSK-FA films. If the exciton power further improved, the third recombination pathway, namely density-dependent non-radiative recombination or Auger recombination, become more and more important and finally dominate, leading to a drop of PLQE. In our research, we focus on the significant enhancement of PLQE for PVSK-FA film, when compared to PVSK film. Notably, the PLQE of PVSK-FA achieved 61% when the excitation power was 100 mW cm^{-2}, which is more than twice as that of PVSK (PLQE = 25%), which confirms the reduction of deep level defect state in PVSK-FA. It correlates to the improvement in V_{OC} in the corresponding device.

Supplementary Note 9.

We consider the discrepancy in iodine management is mostly due to different physiochemical properties in the corresponding absorber layers from the two film growth techniques. More specifically, we consider the major difference in physiochemical properties for two kinds of perovskite films from Yang's work[10] and our study is the defect state properties. In Yang's work, the authors employed deep-level transient spectroscopy (DLTS) and confirmed that the main deep level defects in their fabricated perovskite film was located at energy levels of 0.78 and 0.82 eV below the conduction band, which may be associated with interstitial Pb (Pb_i) and antisite defects. However, in our work, we employed the thermal admittance spectroscopy (TAS) as the main characterization method to determinate the defect state profile, which have been widely used in the organic and perovskite solar cell[11,12]. From the TAS result, we proved that the dominant deep level defect state in our perovskite film has energy level of 0.283 eV above valence band, which might be originated from the interstitial I (I_i). Several published calculation articles have mentioned the amphoteric interstitial I defect can trap both electrons and holes through the +/0 and 0/− ionization levels placed at 0.57 and 0.29 eV below and above the CB and VB edges, respectively[13,14]. For different possible deep level defects, various strategies might been need. Thus, Yang et al reduced the concentration of deep level defects, possibly interstitial Pb, by introduction of iodide ions, and we employed alkaline additives to suppress the incident I_2 in the halide precursor solution and effectively reduce the density of deep level defects, possibly interstitial I, in the resultant films.

In addition, the chemical component (the ratio of FA and MA) for our perovskite film is also quite different with the Yang's perovskite film. And the Yang's perovskite film has more FA component. Our perovskite film contains 5% CsI, which was introduced during the deposition process of PbI_2 film.

Thirdly, we think the major difference in physiochemical properties for two kinds of films was originated from the different film growth techniques. For this point, Yang's group have emphasized the difference between the intramolecular exchange process (IEP, their method) and conventional two-step deposition process (our method) in reported literature[15]. Thus, we won't go into detail of the literature. And we concluded that there are several differences between the conventional two-step process and the intramolecular exchange process, such as the interdiffusion of FAI in pristine PbI_2 film, the insertion of FAI into PbI_2 crystal, and the crystal structure transformation from PbI_2 film (hexagonal phase) to perovskite film (cubic phase).

Considering the second and third point we mentioned above simultaneously, we think all these factors might influence the physiochemical properties for perovskite film and how does the impurity or additives in organic cation precursor solution work when FAI or MAI organic cation reacted with PbI_2 or PbI_2(DMSO) films. Therefore, we think that the approach in our

work just seems contradictory with Yang's work; however, they are quite different due to the different perovskite preparation methods and different perovskite films.

Supplementary Note 10.

We further conducted the PL emission spectra with increasing light intensity by femtosecond laser in PVSK and PVSK-FA films that spin-coated on a quartz substrate, as shown in Supplementary Fig. 20a and 20b. At the low excitation power, the integrated intensity from PVSK and PVSK-FA increases with increasing pump fluence. The different trap saturation threshold fluence (P_{th}^{trap}) have been observed at 1.85 μJ/cm^2 for PVSK and 1.29 μJ/cm^2 for PVSK-FA, as shown in Supplementary Fig. 20c and 20d**.** The lower P_{th}^{trap} indicate that fewer photon was needed to fill the trap state, suggesting lower trap state density. According to the analytical mode presented in previous study[16], we have fitted the experimental data well (R^2 = 0.99). As shown in Supplementary Fig. 20e and 20f**,** the fitted result suggested that the total trap concentration (N_t) in PVSK-FA film is 7.8 (± 0.7) × 10^{16} cm^{-3}, which is lower than the N_t in PVSK film (8.6 (± 0.9) × 10^{16} cm^{-3}).

To characterize the femtosecond optical spectroscopy, A 400 nm laser pulse as the excitation source was obtained by a doubling BBO crystal from the Coherent Astrella amplifier (80 fs, 1 kHz, 800 nm). The pump laser spot size was 250 μm (Objective: 10 ×, NA = 0.3). The signal was collected by the same objective lens into an optical fiber as backscattered emission, which was coupled to a spectrometer (Princeton Instrument SP2500i) and detected by a liquid nitrogen cooled charge coupled device detector. A 442 nm long pass filter was used before the optical fiber to block the pump laser. The optical characterization was conducted at room temperature.

Supplementary Note 11.

In fact, the molar equivalence of additives being added is closely related with the solubility of these alkaline additives in anhydrous ethanol. We have measured the solubility of these alkaline additives in ethanol roughly, and calculated the molar equivalence being added in the organic cation/isopropanol, which is shown in Supplementary Table 4. As expected, the solubility of NaOH and KOH is larger than the other additives, leading to possible precipitation in isopropanol.

To further examine if these precipitations have influence on the morphology of perovskite film rather than possible alkalinity, we have introduced into two other alkaline additives NH_4OH and CH_3NH_3/ethanol solution. These two additives have not caused the precipitation in organic cation/isopropanol. At first, we employed the XRD measurement to confirm that the addition of NH_4OH and CH_3NH_3/ethanol would facilitate the structural transformation from PbI_2 to δ-phase perovskite, just like NaOH and KOH, which is shown in Supplementary Fig. 21a, Meanwhile, the introduction of NaI and KI also would not cause the precipitation in precursor and the generation of δ-phase perovskite due to the lack of strong alkalinity. Then, we further performed the SEM measurement, and corresponding images were shown in Supplementary Fig. 21b-21h. We found that the resultant perovskite film with addition of NaI and KI additives is quite similar with the reference. And the resultant perovskite film with addition of NaOH, KOH, NH_4OH and MA/ethanol solution is different from the reference. Especially, the shape of crystalline grain is various, which might originate from the different phase of perovskite.

Supplementary References

1. Truesdale, V. W., Luther, G. W. & Greenwood, J. E. The kinetics of iodine disproportionation: a system of parallel second-order reactions sustained by a multi-species pre-equilibrium. *Phys. Chem. Chem. Phys.* **5**, 3428–3435 (2003).
2. Wang, S., Jiang, Y., Juarez-Perez, Emilio J., Ono, Luis K. & Qi, Y. Accelerated degradation of methylammonium lead iodide perovskites induced by exposure to iodine vapour. *Nat. Energy* **2**, 16195 (2016).
3. Jiang, Q. *et al.* Planar-Structure Perovskite Solar Cells with Efficiency beyond 21%. *Adv. Mater.* **29**, 1703852 (2017).
4. Chen, Q. *et al.* Controllable self-induced passivation of hybrid lead iodide perovskites toward high performance solar cells. *Nano Lett.* **14**, 4158–4163 (2014).
5. Abdi-Jalebi, M. *et al.* Maximizing and stabilizing luminescence from halide perovskites with potassium passivation. *Nature* **555**, 497–501 (2018).
6. Bu, T. *et al.* A novel quadruple-cation absorber for universal hysteresis elimination for high efficiency and stable perovskite solar cells. *Energy Environ. Sci.* **10**, 2509–2515 (2017).
7. Zheng, G. *et al.* Manipulation of facet orientation in hybrid perovskite polycrystalline films by cation cascade. *Nat. Commun.* **9**, 2793 (2018).
8. Saba, M. *et al.* Correlated electron-hole plasma in organometal perovskites. *Nat. Commun.* **5**, 5049 (2014).
9. Sutter-Fella, C. M. *et al.* High photoluminescence quantum yield in band gap tunable bromide containing mixed halide perovskites. *Nano Lett.* **16**, 800–806 (2016).
10. Yang, W. S., *et al.* Iodide management in formamidinium-lead-halide-based perovskite layers for efficient solar cells. *Science* **356**, 1376–1379 (2017).
11. Ye, S. *et al.* A breakthrough efficiency of 19.9% obtained in inverted perovskite solar cells by using an efficient trap state passivator Cu(thiourea)I. *J. Am. Chem. Soc.* **139**, 7504–7512 (2017).
12. Duan, H. S. *et al.* The identification and characterization of defect states in hybrid organic-inorganic perovskite photovoltaics. *Phys. Chem. Chem. Phys.* **17**, 112–116 (2015).
13. Meggiolaro, D. *et al.* Modeling the Interaction of Molecular Iodine with $MAPbI_3$: A Probe of Lead-Halide Perovskites Defect Chemistry. *ACS Energy Lett.* **3**, 447–451 (2018).
14. Meggiolaro, D. *et al.* Iodine chemistry determines the defect tolerance of lead-halide perovskites. *Energy Environ. Sci.* **11**, 702–713 (2018).
15. Yang, W. S. *et al.* High-performance photovoltaic perovskite layers fabricated through intramolecular exchange. *Science* **348**, 1234–1237 (2015).
16. Xing, G. *et al.* Low-temperature solution-processed wavelength-tunable perovskites for lasing. *Nature Mater.* **13**, 476–480 (2014).
